# MAPK14 converges on key transcriptional machinery to promote vascular smooth muscle cell degeneration in abdominal aortic aneurysm

**DOI:** 10.1038/s41392-025-02540-0

**Published:** 2026-01-12

**Authors:** Xiaoliang Wu, Chunhui Wang, Nestor Ishimwe, Wei Zhang, Jaser Doja, Shengshuai Shan, Chunyu Ge, Yong Sun, Jinjing Zhao, Micah Castillo, Peter Sotonyi, Gergo Gyurok, Gabor Csanyi, W. Bart Bryant, Kunzhe Dong, Yabing Chen, Roberto Vazquez-Padron, Joseph M. Miano, Xiaochun Long

**Affiliations:** 1 Vascular Biology Center, Department of Medicine, Medical College of Georgia, Augusta University, Augusta, GA USA; 2 Department of Pathology & Laboratory Medicine, School of Medicine, Oregon Health & Science University, Portland, OR. Portland Veterans Affairs Medical Center, Portland, OR USA; 3 Department of Molecular and Cellular Physiology, Albany Medical College, Albany, NY USA; 4 Department of Biology and Biochemistry, University of Houston Sequencing and Gene Editing Core, University of Houston, Houston, TX USA; 5 Department of Vascular and Endovascular Surgery, Heart and Vascular Centre, Semmelweis University, 1122 Budapest, Hungary; 6 Vascular Biology Center, Department of Pharmacology & Toxicology, Medical College of Georgia, Augusta University, Augusta, GA USA; 7 Immunology Center of Georgia, Medical College of Georgia, Augusta University, Augusta, GA USA; 8 DeWitt Daughtry Family Department of Surgery, Leonard M. Miller School of Medicine, University of Miami, Florida, USA; 9 Bruce W. Carter Veterans Affairs Medical Center, Miami, FL USA; 10 Present Address: Department of Critical Care Medicine, Ward 3, Shandong Provincial Hospital Affiliated to Shandong First Medical University, Jinan, Shandong China; 11 Present Address: Lemole Center for Integrated Lymphatic and Vascular Research, Lewis Katz School of Medicine, Temple University, Philadelphia, PA USA

**Keywords:** Cell biology, Cardiovascular diseases

## Abstract

Vascular smooth muscle cell (VSMC) degeneration is a major mechanism underlying abdominal aortic aneurysm (AAA) formation. However, the upstream signaling pathways that converge on the transcriptional machinery to drive VSMC degeneration remain elusive. Here, we integrated single-nucleus (sn) multi-omics, chromatin immunoprecipitation (ChIP)-seq, and wet lab validation to identify transcriptional effectors of VSMC-MAPK14, which we previously reported to promote AAA. Compared with wild-type (WT) mice, VSMC-*Mapk14* knockout (KO) mice displayed reduced VSMC degeneration, as evidenced by decreased expression of markers of endoplasmic reticulum stress, the unfolded protein response, fibrosis, and apoptosis, after 7 days of Ang II infusion. SnRNA-seq revealed increased VSMCs and reduced fibroblast and immune cell populations in KOs. Reclustering VSMCs revealed an increased proportion of contractile cluster and a reduced proportion of fibrotic cluster in KOs. The VSMC differentiation gene program and upstream pathways were upregulated, whereas degeneration pathways, including extracellular matrix remodeling, inflammation, and apoptosis, were downregulated in KO VSMCs. snATAC-seq and validation revealed increased serum response factor (SRF) motif activity and expression but reduced RUNX2 expression in KO VSMCs. Integrative analysis of snATAC-seq, ChIP-seq, and bulk RNA-seq identified the MYOCD/SRF/CArG triad as the driver of the contractile gene program following *Mapk14* loss. We further found that the expression of *Bcl2*, a novel MYOCD/SRF/CArG target, was increased in *Mapk14* KO VSMCs. Loss of *Mapk14* attenuated MRTFA protein abundance via increased ubiquitin‒proteasome degradation, which was attributed to reduced USP10 protein expression. These findings reveal MAPK14-driven transcriptomic and epigenomic landscapes that promote VSMC degeneration by suppressing SRF/MYOCD/CArG while activating RUNX2 and MRTFA. Our study provides mechanistic insight into MAPK14-mediated VSMC degeneration and provides a basis for MAPK14-targeted therapeutic strategies for AAA.

## Introduction

Abdominal aortic aneurysm (AAA), characterized by progressive dilatation and weakening of the abdominal aorta (AA), is a devastating disease associated with high morbidity and mortality due to fatal dissection and rupture.^1,2^ Despite decades of investigation, no effective pharmacological therapies exist for AAA. Although extensive evidence supports the crucial role of vascular smooth muscle cell (VSMC) degeneration, a process in which contractile VSMCs phenotypically switch to proinflammatory, fibrotic, apoptotic, and senescent state(s) during AAA development, the underlying molecular mechanisms driving VSMC degeneration, particularly how upstream signaling pathways converge on the transcriptional machinery, remain incompletely understood.

Contractile VSMCs are genetically programmed to maintain their primary function, ensuring mechanical strength, elasticity, and contractility through a repertoire of genes encoding cytocontractile proteins, ion channel subunits, and noncoding RNAs.^3–9^ The transcription of these genes is governed primarily by a master switch composed of the potent cofactor, Myocardin (MYOCD), the associated DNA-binding transcription factor (TF) serum response factor (SRF) and its *cis* element, called CArG box.^10,11^ On the other hand, VSMCs possess a remarkable capacity to switch their original contractile and quiescent phenotype to proinflammatory, fibrotic, chondrogenic, and osteogenic states in response to biomechanical and biochemical stresses.^12,13^ While such transient phenotypic switching serves as a protective adaptation to preserve vascular integrity under stress, excessive VSMC transdifferentiation reduces vessel contractility and elasticity and causes structural deterioration, ultimately contributing to aortic destruction and AAA formation. Regardless of the specific phenotypic outcome, a prerequisite for VSMC phenotypic switching is VSMC dedifferentiation, characterized by reduced expression of contractile genes. This process is largely mediated by the perturbation of the MYOCD/SRF/CArG transcriptional triad via reduced levels of the MYOCD cofactor.^14,15^ Although the lack of reliable MYOCD antibodies has hindered the direct investigation of MYOCD protein expression,^16^ reduced *Myocd* mRNA has been consistently linked to vascular degeneration and aortic aneurysm.^15,17^ In contrast, MRTFA, a paralog of MYOCD,^18^ is increased in vascular disease and has emerged as a critical driver of various vascular complications, including vascular stenosis, atherosclerosis, and aortic aneurysm.^19–21^ Notably, the therapeutic potential of targeting MRTFA is supported by preclinical studies showing that selective MRTFA inhibitors effectively prevent vascular disease progression.^19,22^ Despite the growing recognition of the distinct regulation and functional roles of MYOCD and MRTFA in vascular pathology, it remains unclear whether these two factors are governed by a shared upstream signaling pathway that orchestrates VSMC degeneration and AAA formation.

MAPK14, which encodes p38α, the predominant isoform of the MAPK family in VSMCs, serves as a convergence point for diverse pathological stimuli, including mechanical strain, oxidative stress, and inflammatory signaling.^23,24^ Through this central role, MAPK14 governs key pathological processes such as inflammation, cell proliferation, senescence, and apoptosis. As such, MAPK14 acts as a potential modulator of the VSMC phenotype. While targeting the broader p38 MAPK family with selective inhibitors has shown limited therapeutic promise due to off-target effects,^25^ specifically targeting MAPK14 in VSMCs using genetic animal models has demonstrated consistent protection against various vascular complications, including injury-induced vascular stenosis, atherosclerosis, and aneurysm formation, underscoring its therapeutic potential in vascular disease.^19,26,27^ Our recent studies revealed that VSMC-specific depletion of *Mapk14* prevents AAA formation, which is accompanied by reduced VSMC inflammation and senescence.^19^ Although activation of MAPK14 is essential for mediating the functional consequence of MRTFA in aortic aneurysm, the mechanisms through which MAPK14 intersects with key transcriptional effectors in AAA formation remain unexplored.

The advent of single-nucleus RNA sequencing (snRNA-seq) and ATAC sequencing (snATAC-seq) has provided unprecedented resolution for dissecting cellular heterogeneity and gene regulatory networks.^28–30^ Integration of snRNA-seq and ATAC-seq enables the identification of key transcriptional regulators and their downstream targets. In this study, we integrated sn multi-omics, chromatin immunoprecipitation (ChIP)-seq, and wet-lab mechanistic validation to define the transcriptomic and epigenomic landscapes regulated by VSMC-MAPK14. Our findings reveal, for the first time, that VSMC-MAPK14 signaling converges on opposing transcriptional effectors, MYOCD/SRF versus MRTFA and RUNX2, which together drive VSMC degeneration and AAA formation. These results establish a molecular framework linking MAPK14 signaling to key TFs and highlight their combined functional significance in promoting VSMC degeneration and AAA formation.

## Results

### Loss of MAPK14 in VSMCs antagonizes VSMC degeneration in an Ang II-induced AAA model

To systematically characterize the dynamic fate and state of VSMCs during AAA formation, we isolated AAs from *Apoe*^*-/-*^ mice at different time points after Ang II infusion and subjected them to different assays (Supplementary Fig. 1a). Western blot analysis revealed a sharp reduction in MYH11 and LMOD1 in AAs at 7, 14, and 21 days after Ang II infusion. In contrast, the expression of endoplasmic reticulum (ER) and unfolded protein response (UPR) chaperone proteins, such as CALR, P4HB, HSPA5, spliced XBP1 (XBP-1s), and the stress TFs ATF3 and ATF6, as well as the apoptosis marker cleaved caspase 3 (cl-CASP), markedly increased over this time course (Supplementary Fig. 1b, c). To determine whether such changes occurred in medial VSMCs, we conducted co-immunofluorescence staining for P4HB and the VSMC marker ACTA2. Consistent with the Western blot results, while strong ACTA2 but undetectable P4HB was detected in the AAs prior to Ang II infusion (day 0), a significant reduction in ACTA2 coincided with an increase in P4HB on day 7 and day 21 after Ang II infusion (Supplementary Fig. 1d, e). The strong colocalization of ACTA2 with P4HB indicates VSMC-specific induction of P4HB. A similar reduction in MYH11 in AAs was observed after Ang II infusion (Supplementary Fig. 1d, e). Excessive reactive oxygen species (ROS) plays a critical role in the initiation and progression of aortic aneurysm, which can be frequently demonstrated with dihydroethidium (DHE) staining.^31^ Virtually undetectable DHE staining was observed in day 0 vessels, but a progressive increase in DHE staining was observed in day 7 and day 21 vessels (Supplementary Fig. 1d, e). Importantly, these changes were accompanied by the induction of pMAPK14, as revealed by immunohistochemical staining after Ang II infusion for 7 and 21 days (Supplementary Fig. 2a, b). Consistently, human AAA medial layer cells presented increased levels of pMAPK14, whereas the non-AAA control did not display any detectable pMAPK14 signal (Supplementary Fig. 2c, d). These results demonstrate that VSMC degeneration precedes Ang II-induced AAA formation.

**Fig. 1:**
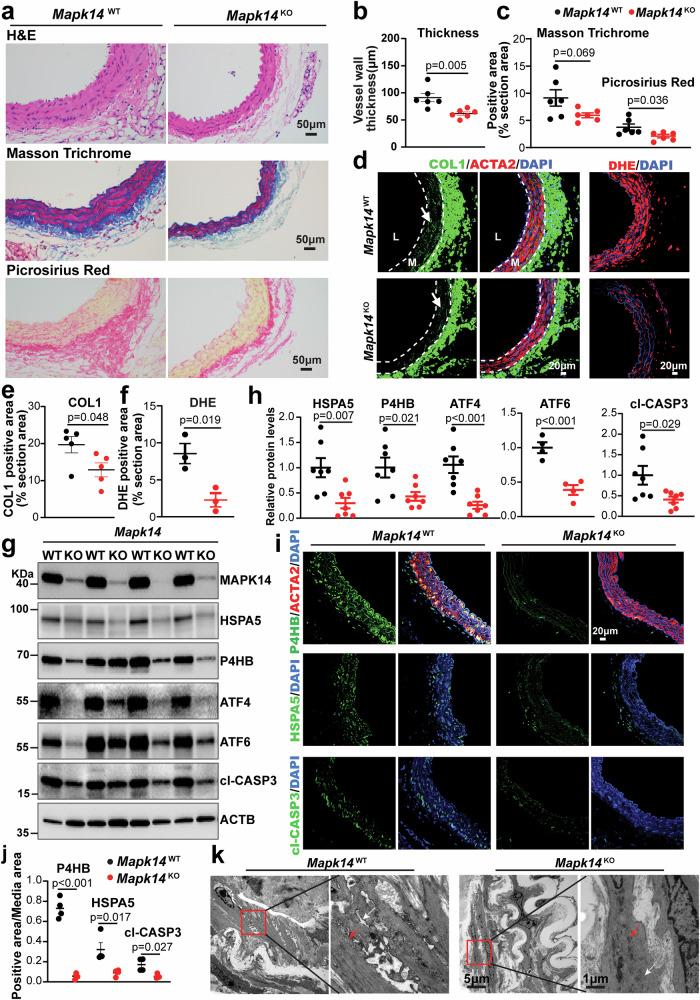
Deficiency of MAPK14 in VSMCs impedes Ang II-induced aortic remodeling and VSMC degeneration. *Mapk14*^WT^ (*Mapk14*^*f/f*^/*Apoe*^-/-^) and *Mapk14*^KO^ (*Sm22-*Cre^+/-^/*Mapk14*^*f/f*^*/Apoe*^-/-^) mice were infused with Angiotensin II (Ang II) for 7 days, and abdominal aortas (AAs) were isolated for the following experiments. **a** Representative images of hematoxylin and eosin (H&E), Masson’s trichrome, and Picrosirius Red staining of cross sections of AAs from the indicated mice. **b** Quantification of vessel wall thickness (medial and adventitial layers) via H&E staining (*n* = 6 per group). **c** Quantification of fibrosis via Masson’s trichrome and Picrosirius Red staining (*n* = 6 per group). **d** Representative immunofluorescence images of COL1 (left) and dihydroethidium (DHE) staining (right) in AAs from the indicated mice. **e**, **f** Quantification of the COL1-positive area (**e**) and DHE positive area (**f**) in AAs (*n* = 6 per group). **g** Representative Western blot images of the indicated proteins in AAs from the indicated mice. **h** Quantification of the levels of the indicated proteins in the indicated groups (*n* = 7 per group for HSPA5, PBH4, ATF4, and cl-CASP3; *n* = 4 per group for ATF6). **i** Representative immunofluorescence images showing PBH4, HSPA5, cl-CASP3 (green), ACTA2 (red), and DAPI (blue) in cross-sections of AAs. **j** Quantification of the positive area of the indicated proteins within the media layer of AAs (*n* = 4 per group). **k** Representative transmission electron microscopy (TEM) images showing swollen mitochondria and enlarged endoplasmic reticulum (ER) in *Mapk14*^WT^ compared with *Mapk14*^KO^ mice (*n* = 3 per group). Red arrows indicate swollen mitochondria, and white arrows indicate enlarged ER. The data were analyzed via Student’s *t* tests for all quantifications. L lumen; M medial layer

We previously reported that the loss of MAPK14 in VSMCs markedly prevented aortic dissection and AAA formation in an Ang II-induced mouse AAA model.^19^ To determine the precise mechanism underlying the protection exerted by MAPK14 loss in VSMCs, we isolated AAs from *Mapk14* KO (*Sm22-Cre/Mapk14*^f/f^/*Apoe*^-/-^, hereafter referred to as KO) and WT littermate (*Mapk14*^f/f^/*Apoe*^-/-^, hereafter referred to as WT) mice on day 7 after Ang II infusion, an early stage of AAA development. At this stage, KO AAs presented significantly reduced medial and adventitial thickness, along with decreased fibrosis, as demonstrated by hematoxylin and eosin (H&E) and Picrosirius Red staining. Masson’s trichrome staining revealed a similar trend of reduced fibrosis, although the reduction was statistically insignificant (Fig. 1a–c). Costaining for COL1 and ACTA2 revealed reduced COL1 accumulation in both the medial layer and the adventitia in KO AAs, with the latter being negative for ACTA2 (Fig. 1d, e). Furthermore, the KO vascular wall, particularly the adventitia, exhibited significantly decreased cell proliferation and inflammatory cell infiltration, as indicated by Ki67, CD68, and LGALS3 staining (Supplementary Fig. 3a–f). On the other hand, depletion of MAPK14 significantly decreased TNFα-induced MAPK14 activation, as evidenced by reduced levels of pMAPK14, which was accompanied by significantly decreased levels of secreted IL6 (Supplementary Fig. 3g–i). These findings suggest that VSMC-MAPK14 drives adventitial remodeling during AAA formation through paracrine signaling from medial VSMCs. Previous studies using a selective p38MAPK inhibitor reported a crucial role of this pathway in increasing oxidative stress.^32^ Consistently, DHE staining revealed a drastic reduction in oxidative stress in the medial VSMC layer of the KO AAs (Fig. 1d, f).

Increased ER stress and the unfolded protein response (UPR) have emerged as driving forces for VSMC phenotypic transition and cell death in vascular disease.^33,34^ While the expression of ER/UPR markers increased during Ang II-induced AAA development (Supplementary Fig. 1d, e), Western blot analysis revealed a sharp downregulation of the expression of HSPA5, P4HB, ATF4, and ATF6, as well as the apoptosis marker cleaved caspase-3 (cl-CASP3), in KOs (Fig. 1g, h). The reduced VSMC apoptosis in AAs from KOs was further validated by costaining with TUNEL and the VSMC marker ACTA2 (Supplementary Fig. 4a, b). Immunostaining further confirmed a reduction in P4HB, HSPA5, and cl-CASP3 in medial SMCs in KOs (Fig. 1i, j), whereas no significant changes in ER stress proteins were detected between WT and KO saline-infused mice (Supplementary Fig. 5a, b). To determine the ultrastructural differences in VSMCs between WT and KO mice, we performed transmission electron microscopy (TEM) on AAs from both groups infused with Ang II for 7 days. Although the abundance of myofilaments in WT and KO AAs was indistinguishable, the size of VSMCs was much smaller in KO mice than in WT mice, suggesting a potential role of MAPK14 in VSMC hypertrophy. While substantially swollen ER lumina and loss of mitochondrial contents were widespread in VSMCs from WT mice, the integrity of the ER and mitochondria was more preserved in KOs (Fig. 1k). Collectively, these results demonstrate a previously unrecognized role for MAPK14 loss in preventing VSMC degeneration manifested by attenuation of adventitial fibrosis and ER stress, oxidative stress, and apoptosis in aortic medial VSMCs.

### snRNA-seq reveals transcriptomic changes that preserve vascular integrity upon VSMC-specific loss of MAPK14 during AAA formation

**Fig. 2:**
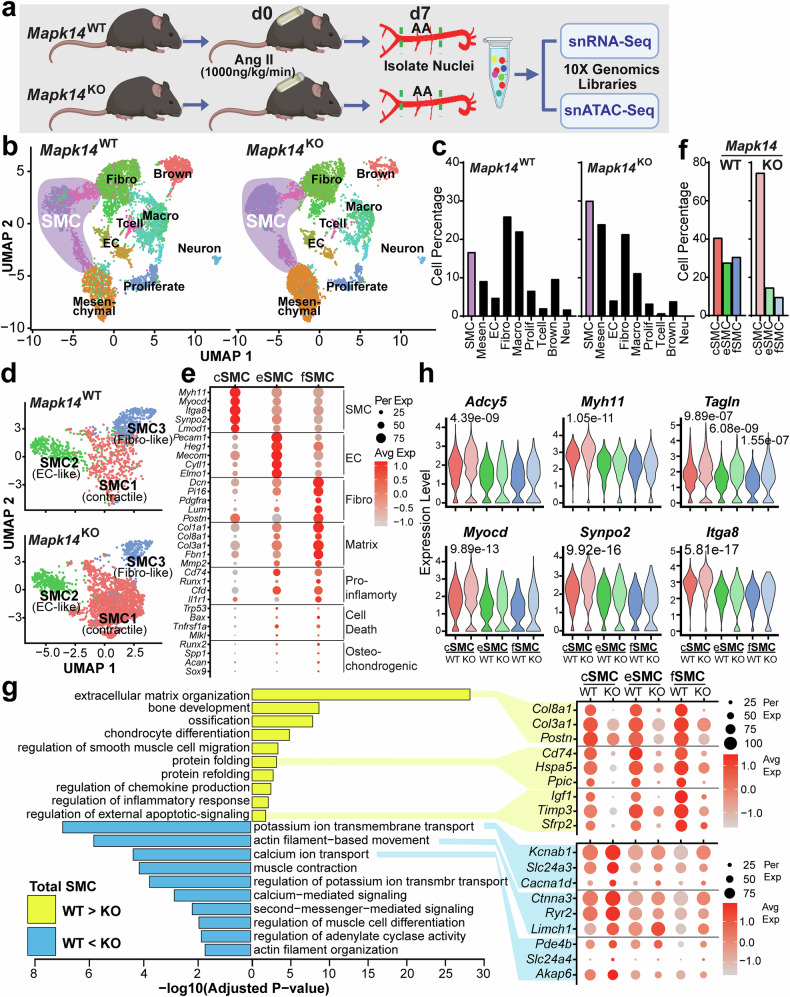
snRNA-seq reveals transcriptomic changes that preserve vascular integrity upon VSMC-specific loss of MAPK14 during AAA formation. **a** Schematic of the workflow for snRNA-seq and ATAC-seq for AAs isolated from *Mapk14*^*f/f*^/*Apoe*^-/-^ (*Mapk14*^WT^) and *Sm22-*Cre^+/-^/*Mapk14*^*f/f*^*/Apoe*^-/-^ (*Mapk14*^KO^) mice infused with Ang II for 7 days (*n* = 3 per reaction) (created with https://www.biorender.com/). **b** Uniform manifold approximation and projection (UMAP) visualization of single nuclei. Each dot represents one nucleus, and all the nuclei are colored according to the cluster identification. **c** A bar plot showing the percentage of each identified cell cluster in the *Mapk14*^WT^ and *Mapk14*^KO^ groups. **d** UMAP visualization of smooth muscle cell (SMC) subpopulations. **e** Dot plots showing the expression of marker genes of VSMCs, ECs, and fibroblasts and the indicated degenerative gene programs in the three SMC clusters. Dot size and color indicate the proportion of cells expressing each gene and the level of expression, respectively. A deeper color denotes higher expression, whereas a lighter color denotes lower expression. The average expression scale is displayed on the right. **f** Bar plot showing the relative proportions of each SMC subcluster within the SMC population. **g** Bar plots showing the significantly changed pathways revealed by clusterProfiler within the total SMC population; the top enriched pathways upregulated in *Mapk14*^WT^ (yellow) and *Mapk14*^KO^ (blue) SMCs (left) are shown. Gene expression of the relevant candidate genes for the indicated pathways in each SMC cluster is shown in dot plots (right). **h** Violin plot showing the expression of contractile VSMC markers in each of the three SMC clusters

To elucidate the mechanism through which MAPK14 promotes VSMC degeneration, we performed snRNA-seq and snATAC-seq experiments to define the transcriptomic and epigenetic profiles at single-cell resolution. To this end, we extracted nuclei from the AAs of 12-week-old male *Mapk14*^WT^ and *Mapk14*^KO^ mice infused with Ang II for 7 days. Approximately 10,000 high-quality AA nuclei derived from 3 animals per group were processed for the multiome ATAC and gene expression libraries via the 10x Genomics platform (Fig. 2a). For the snRNA-seq libraries, doublets and low-quality nuclei were removed via single-cell TK^35^ and quality control via Seurat,^36^ resulting in 7284 and 7811 high-quality nuclei for the WT and KO groups, respectively.

Unsupervised clustering and cell type nomenclature based on known cell markers per cell type revealed nine cell populations, including VSMCs (SMC), mesenchymal cells (Mesenchymal), endothelial cells (EC), fibroblasts (Fibro), macrophages (Macro), T cells, neurons, Brown adipocytes (Brown), and a highly proliferative mixed cell cluster (Proliferate) (Fig. 2b, Supplementary Fig. 6a). The mesenchymal cell cluster lacks gold standard VSMC markers, such as *Myocd*, *Lmod1*, *Carmn*, and much lower *Myh11* but expresses high levels of mesenchymal markers, such as *Vim*, *Fn1*, and *Acta2* (Supplementary Fig. 6b). Because these cells express the specific EC marker *Cdh5* and high levels of the arterial EC marker *Gja5*, this cell cluster is likely derived from neovascularization during AAA formation. The proportions of VSMCs and mesenchymal cells were higher in KO mice (30% and 25%) compared with WT mice (17% and 9%). (Fig. 2c). While the proportion of total ECs remained largely the same in both groups, virtually all other cell types, including Fibro-, Macro-, Proliferate-, T-cell-, and Brown-cell clusters, were reduced to varying degrees in KO mice compared with WT mice (Fig. 2c).

To investigate the specific impact of MAPK14 on VSMC heterogeneity, we subset the “classical” SMCs into a VSMC-specific nonlinear dimensional reduction, which yielded three unique VSMC clusters (Fig. 2d). A profile of the top five differentially expressed genes (DEGs) revealed three distinct VSMC clusters. Contractile SMC1 (cSMC) displayed the highest levels of contractile markers (*Myh11, Myocd, Itga8*), whereas EC-like SMC2 (eSMC) was characterized by endothelial genes (*Pecam1*, *Heg1*), and fibroblast-like SMC3 (fSMC) showed high levels of fibroblast markers (*Dcn, Pi16, Pdgfra*) (Fig. 2e, Supplementary Fig. 6c). SMC2 was different from the mesenchymal cell cluster, as the latter had lost mature SMC markers (Supplementary Fig. 6b). SMC1 is the dominant VSMC population in both WT and KO AA samples. Its proportion within the total SMC population was significantly higher in the KO (75%) compared with the WT (41%). In contrast, SMC2 and SMC3 subclusters were more prevalent in WT mice, accounting for 28% and 31%, respectively, but were reduced to 15% and 10% in KO mice (Fig. 2f). In addition, SMC2 and SMC3 exhibited relatively high expression of matrix (*Col1a1, Col8a1, Col3a1*), proinflammatory (*Cd74*, *Runx1*, *Cfd*), cell death-associated (*Bax*, *Tnfrsf1a*, *Mlkl*), and osteochondrogenic (*Runx2*, *Spp1*, *Sox9*) genes (Fig. 2e). We therefore consider SMC2 and SMC3 to be degenerative SMC clusters.

To determine the impact of the loss of MAPK14 function, specifically in SMCs, on AAA formation, we used the R package clusterProfiler^37^ to perform pathway enrichment analysis of the snRNA-seq gene expression data. As expected, extracellular matrix organization was one of the top upregulated pathways in the WT (Fig. 2g). Additionally, other pathological pathways, including bone development, calcification, chondrogenesis, protein folding, inflammation, and apoptosis, were also upregulated in the SMC populations from the WT compared with their KO counterparts (Fig. 2g). In contrast, pathways upregulated in SMCs from the KO group compared with those from the WT group included potassium ion transmembrane transport, actin filaments, calcium ion transport, muscle contraction, SMC differentiation, second messenger signaling, and calcium-mediated signaling (Fig. 2g). To illustrate the contributions of genes to pathways identified by clusterProfiler, we plotted the expression of the relevant gene sets via dot plots to illustrate the dichotomy of the above significantly changed biological processes in all three SMCs between the WT and KO groups. These genes included highly downregulated matrix, protein folding, and apoptotic genes and upregulated potassium ion transmembrane transport, actin filament-based movement, and calcium ion transport genes across all three SMC clusters in the KO group compared with those in the WT group (Fig. 2g). Finally, violin plots revealed that the second messenger *Adcy5* and contractile genes, such as *Myh11*, *Tagln*, *Itga8*, and *Synpo2*, and the master regulator of VSMC differentiation, *Myocd*, were also significantly increased in the major SMC cluster, SMC1, in KO compared with WT mice (Fig. 2h). Together, these results demonstrate that loss of MAPK14 protects aortic wall integrity by maintaining VSMC contents and reducing immune inflammatory cell infiltration, as well as safeguarding VSMC differentiation while antagonizing VSMC degenerative alterations.

### snATAC-seq reveals distinct epigenomic profiles in AAs from *Mapk14* WT and KO mice during AAA formation

**Fig. 3:**
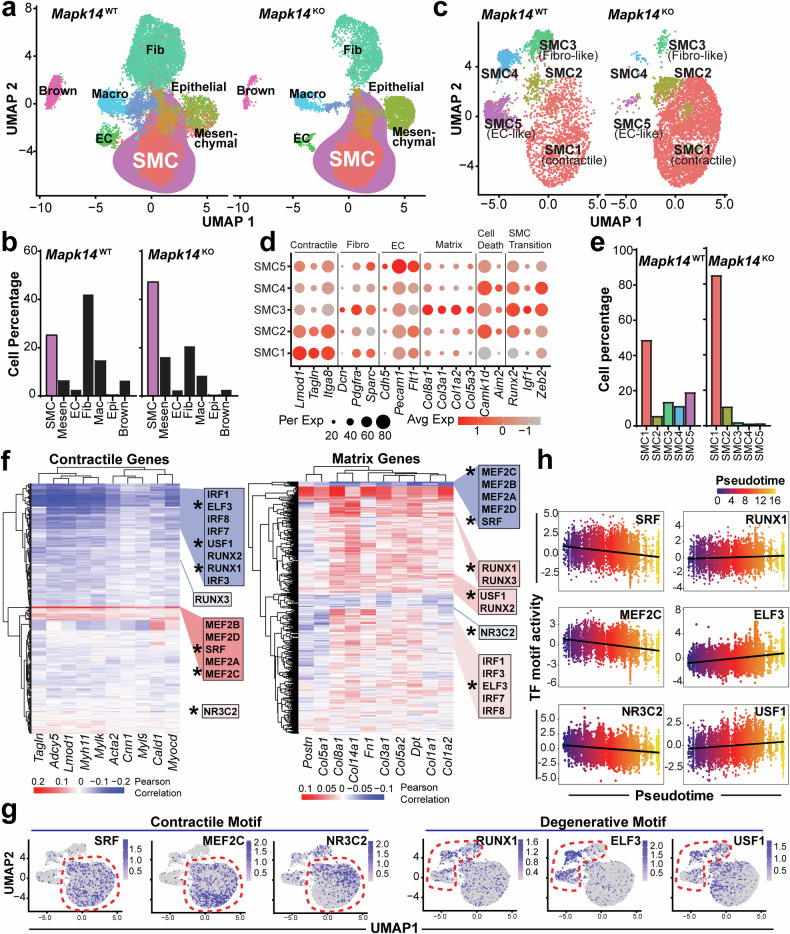
snATAC-seq reveals distinct epigenomic profiles in AAs from *Mapk14* WT and KO mice during AAA formation. **a** Uniform manifold approximation and projection (UMAP) visualization of single nuclei from the snATAC-seq data. Each point represents a single nucleus, and all nuclei are colored according to the cluster identification. **b** A bar plot showing the percentage of each identified cell cluster to the total number of AA cells from *Mapk14*^WT^ and *Mapk14*^KO^ mice. **c** UMAP visualization of the smooth muscle cell (SMC) subclusters identified from the snATAC-seq data. **d** Dot plots showing the indicated VSMC, EC, and fibroblast markers and the indicated degenerative genes in the five identified SMC subclusters based on gene activity derived from snATAC-seq data. Dot size and color indicate the proportion of cells and the level of gene activity, respectively. A deeper color denotes higher gene activity, whereas a lighter color denotes lower gene activity. **e** Bar plot showing the percentage of each SMC subcluster among the total SMCs in the AAs from *Mapk14*^WT^ versus *Mapk14*^KO^ mice. **f** Heatmap showing correlation coefficients between the gene activity of contractile genes (left) and matrix remodeling genes (right) and the motif activity of key transcription factors (TFs). **g** Feature plot visualization of motif activity of the major TFs enriched in contractile (left) and degenerative (right) SMC clusters revealed by chromVAR. **h** Motif activity dynamics of the TF of interest along pseudotime derived from snATAC-seq data. The x-axis represents pseudotime progression, which transitions from early (purple) to late (yellow) stages, whereas the y-axis indicates TF motif activity scores

For the snATAC-seq libraries, doublet and low-quality nuclei were excluded via singleCellTK^35^ and quality control via Seurat,^36^ yielding 12,318 and 8531 high-quality nuclei for the WT and KO snATAC-seq libraries, respectively. To analyze single-cell chromatin accessibility as a proxy for TF activity, the R package Signac was used.^38^ Signac utilizes Cicero^39^ and chromVar^40^ to infer “gene activity” and “motif activity” per cell, respectively. Additionally, Signac seamlessly integrates with Seurat functions, enabling streamlined multimodal analysis. Unsupervised clustering of the snATAC-seq data identified seven cell clusters. To annotate these clusters, we integrated the matched snRNA-seq dataset using Signac and applied Seurat’s label transfer workflow to assign cell identities to the snATAC-seq clusters (Fig. 3a). Surprisingly, the Proliferate, T cell, and Neuron cell clusters detected by snRNA-seq were not captured in snATAC-seq dataset. Conversely, a small population of “epithelial” cells detected in snATAC-seq dataset was not captured by the snRNA-seq (Figs. 2b and 3a). Despite these discrepancies in minor cell populations, changes in the major cell clusters were captured in the snATAC-seq assay, including greatly increased proportions of SMC and mesenchymal populations, comparable proportions of ECs, and reduced proportions of fibroblasts, macrophages, and brown adipocytes in the KO group relative to the WT group (Fig. 3a, b). These patterns were consistent with the snRNA-seq data. To investigate the specific influence of VSMC-MAPK14 on the VSMC population in the ATAC-seq dataset, we first subset the SMC clusters, which yielded five distinct cell clusters (Fig. 3c). When gene activity was assessed from the snATAC-seq data, the notable attributes per cluster illustrated the heterogeneity of SMCs in this context. Similar to snRNA-seq, snATAC-seq captured the contractile SMC1, which exhibited the highest gene activity for contractile genes (*Tagln*, *Lmod1*, *Itga8*) and the lowest gene activity for degenerative genes, including matrix genes (*Col8a1, Col3a1, Col5a3*), cell death-associated genes (*Camkd1d, Aim2*), and activators of VSMC phenotypic modulation (*Runx2, Igf1, Zeb2*) (Fig. 3d). SMC2 retained some of the activity of VSMC contractile genes but also increased the activity of cell death genes (Fig. 3d, Supplementary Fig. 7). SMC3, SMC4, and SMC5 represented more de-differentiated SMC clusters, each showing markedly reduced contractile gene activity. Among these, SMC3 showed the highest gene activity for fibrotic genes and was therefore designated as the fibro-SMC (Fig. 3d). SMC4 displayed strong activity of cell death and SMC transition genes, whereas EC-like SMC5 showed high activity of endothelial genes (*Cdh5, Pecam1, Flit1*) alongside proliferative genes (*Ptprb*, *Bmp6*) (Fig. 3d, Supplementary Fig. 7). Because SMC3, SMC4, and SMC5 are more dedifferentiated and display higher gene activity of degenerative markers, we considered them to be degenerative SMC clusters. Notably, the increased size of contractile SMC1 cluster and reduced size of degenerative SMC3, SMC4, and SMC5 clusters in KO mice were consistent with the snRNA-seq results (Figs. 2f and 3e).

To identify the potential TFs that drive gene networks relevant to VSMC phenotypes, we calculated Pearson correlation coefficients between Signac’s ‘gene activity’ and ‘motif activity’ assays. As proof of principle, the motif activity of SRF and MEF2 family members was strongly positively correlated with the gene activity of SMC contractile genes (Fig. 3f, *left*) and negatively correlated with the gene activity of matrix genes (Fig. 3f, *right*), which is consistent with the known functions of the SRF and MEF2 families in driving the VSMC contractile gene program and the published snATAC-seq data in human vessels.^41^ In contrast, the motif activities of RUNX TF family members (RUNX1-3) and IRF family members (IRF1,3, 7, 8), which are known to promote VSMC phenotypic alterations,^42,43^ and ELF1 were negatively correlated with contractile gene activity but positively correlated with matrix gene activity (Fig. 3f, *right*). Motif activity UMAP feature plots of these TFs revealed that SMC clusters exhibited a dichotomy between contractile and degenerative cell states (Fig. 3g). The motif activity of SRF and MEF2C was enriched in the more contractile SMC1 and SMC2 clusters, whereas RUNX1 and ELF3 ‘motif activity’ was enriched in the more degenerative SMC3, SMC4, and SMC5 clusters (Fig. 3g). Additionally, the motif activity of NR3C2 and USF1, which are reported to regulate blood pressure^44^ and vascular calcification,^45^ respectively, also exhibited inverse associations with contractile and degenerative VSMC phenotypes. Specifically, NR3C2 ‘motif activity’ was enriched in the contractile state, whereas USF1 was enriched in the degenerative VSMC state (Fig. 3g). To gain insight into the dynamic transitions among different SMC clusters, we performed pseudotime trajectory analysis based on the motif activity of the aforementioned TFs. This analysis revealed that the motif activity of SRF, MEF2C, and NR3C2 gradually reduced whereas that of RUNX1, ELF3, and USF1 increased from SMC1 to SMC5 (Fig. 3h), reinforcing the potential role of these TFs in inversely regulating the VSMC contractile and degenerative phenotypes. Collectively, these results further support the heterogeneity of VSMCs at the epigenetic level and highlight potentially key TFs that regulate the contractile and degenerative phenotypes of VSMCs.

### Loss of MAPK14 triggers a distinct epigenetic profile associated with the activation of the contractile but suppression of degenerative transcription machinery

**Fig. 4:**
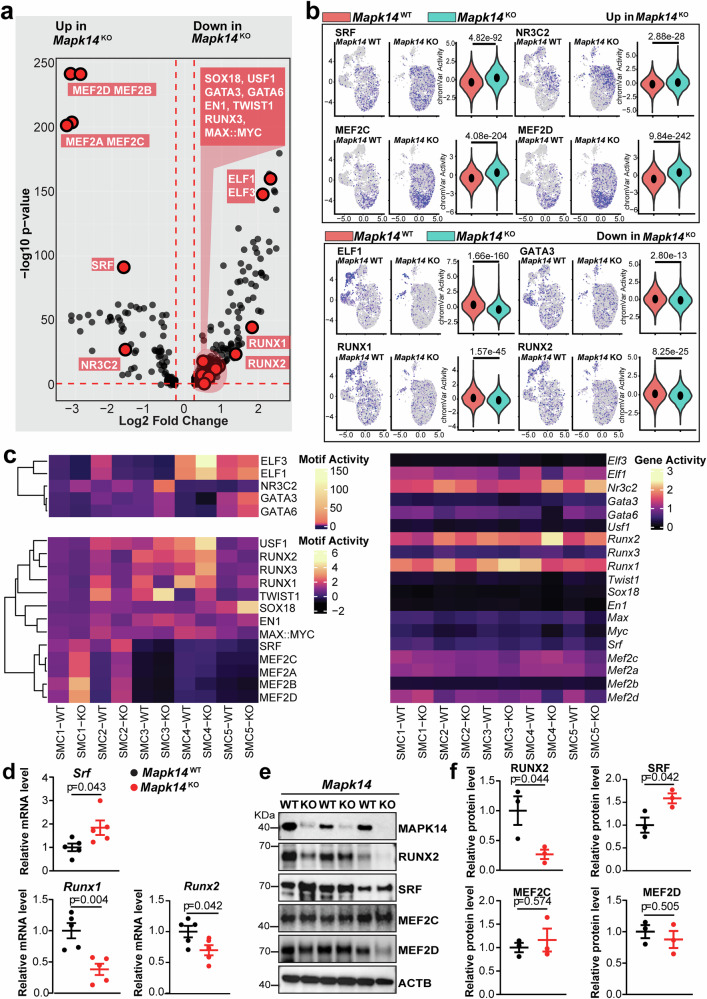
Loss of MAPK14 triggers a distinct epigenetic profile associated with the activation of the contractile but suppression of degenerative transcription machinery. **a** Volcano plot showing the significant motif activity of transcription factors (TFs) associated with VSMC contractile and degenerative gene programs. The X-axis represents the log2-fold change, whereas the Y-axis represents the -log10 *p* value. Red-labeled TFs indicate significantly upregulated or downregulated motifs in *Mapk14* ^KO^ VSMCs. The red dashed lines denote statistically significant thresholds. **b** Feature plots showing the motif activity of TFs (enriched in the contractile SMC cluster) across five SMC subclusters (left) and a violin plot depicting the motif activity of these TFs in the total SMC population (right) in AAs from *Mapk14*^WT^ and *Mapk14*^KO^ mice. **c** Heatmap with hierarchical clustering showing motif activity (left) and gene activity (right) of representative TFs across the five SMC clusters from *Mapk14*^WT^ and *Mapk14*^KO^ mice. **d** qRT‒PCR analysis of the indicated TFs in AAs from *Mapk14*^WT^ and *Mapk14*^KO^ mice infused with Ang II for 7 days (*n* = 5 per group). **e** Western blot analysis of the indicated TFs in AAs from *Mapk14*^WT^ and *Mapk14*^KO^ mice. **f** Quantification of the indicated protein levels (*n* = 3 per group). The data were analyzed using Student’s *t* tests for (**d**, **f**)

After using ‘motif activity’ to identify TF candidates that define either the contractile or degenerative VSMC phenotype, we next investigated the role of MAPK14 in regulating TF motif activity across various cell clusters. We first collapsed all VSMC cell clusters into one single group to compare ‘motif activity’ across genotypes, which resulted in an overarching unbiased view of the snATAC-seq dataset (Fig. 4a). Overall, this comparison revealed that contractile-associated TFs, such as SRF and MEF2 members, and degenerative-associated TFs, such as RUNX and ELF members, were up- and downregulated in *Mapk14* KO, respectively (Fig. 4a). This cell state bifurcation identified in the snATAC-seq data is consistent with the upregulated VSMC differentiation and downregulated matrix organization pathways in VSMCs from the KO group revealed by the snRNA-seq assay (Fig. 2g, h). The motif activity UMAP feature plots of the TFs mentioned above in different SMC clusters further highlight the inverse correlation of the TFs in both groups (Fig. 4b). In addition, UMAP plots revealed that the motif activity of SRF, MEF2 family members (MEF2C and MEF2D), and NR3C2 was increased in the more contractile SMC1 and SMC2 clusters. Moreover, ELF1, GATA3 (tumor metastasis-associated factor),^46^ and RUNX family members (RUNX1 and RUNX2) were downregulated in the more degenerative SMC3, SMC4, and SMC5 clusters of the KO VSMCs (Fig. 4b). A violin plot of motif activity for the above TFs within the total SMC population further confirmed the upregulation of contractile-associated TFs and the downregulation of degenerative-associated TFs in SMCs from KOs (Fig. 4b).

To query motif activity across both the SMC cluster and the genotype variables, a heatmap with hierarchical clustering of TF motif activity was generated (Fig. 4c, *left*). As TF motif activity alone is not conclusive and the abundance of TFs is essential for ultimate TF transcription activity, a gene activity heatmap of the corresponding TFs was generated to support the findings (Fig. 4c, *right*). It should be noted, the heatmap illustrates motif activity per cell cluster per genotype, whereas the volcano plot illustrates motif activity across all VSMC clusters combined per genotype. This point is particularly important given that SMC1 accounts for more than 80% of KO VSMCs (Fig. 3e). Contractile-associated TFs, including SRF, MEF2 family members, and NR3C2, were upregulated in the SMC1 and SMC2 clusters in the KO group (Fig. 4c, *left*). Among these, only NR3C2 exhibited activity beyond SMC1 and SMC2, with a slight increase in motif activity in KO SMC3 (Fig. 4c, *left*). Degenerative-associated TFs, including ELF and RUNX family members, exhibited various trends across the VSMC clusters. ELF1 and ELF3 were upregulated in SMC4 and SMC5 in the KO group, which contrasts with their overall downregulation in KO VSMCs (Fig. 4a, c, *left*). Notably, SMC3, SMC4, and SMC5 represent a small fraction of the total VSMC population in the KO group (Fig. 3e), which explains the overall downregulation of motif activity for specific TFs due to low cell abundance. Cross-referencing motif activity and gene activity heatmaps enabled the identification of TFs that contributed to the *Mapk14* KO phenotype. For example, while ELF1 and ELF3 exhibited high motif activity in the SMC3 and SMC4 clusters, ELF3 showed sparse gene activity in all five SMC clusters (Fig. 4c). A similar trend was observed for the RUNX family members, among which RUNX3 displayed the lowest gene activity. Additionally, despite the various known roles of USF1, SOX18, EN1, and MEF2B in vascular contexts, none demonstrated strong gene activity in our dataset (Fig. 4c, *right*). Cross-referencing motif and gene activity assays helped narrow down biologically relevant TFs to further query. For example, although pseudotime analysis suggested a role for USF1 in the VSMC degenerative cell state (Fig. 3h), its lack of gene expression (Fig. 4, *right*) contradicts this interpretation. Finally, cross-referencing these assays in our snATAC-seq dataset revealed that, while SRF, MEF2 family members, and NR3C2 showed broad expression across all VSMC clusters in the KO group, SRF exhibited more consistent increases in both motif and gene activity in the SMC1 and SMC2 clusters in the KO group, suggesting its functional importance in mediating the contractile phenotype caused by MAPK14 loss in VSMCs (Fig. 4c). Conversely, although all the RUNX members exhibited reduced motif and gene activity in SMC1 and SMC2, the gene activity of RUNX3 was considerably low across all the SMC clusters, and RUNX2 displayed a more consistent decrease in both motif and gene activity across all SMC clusters in the KO group (Fig. 4c). These analyses suggest the potential roles of SRF, MEF2C/D, and RUNX1/2 in mediating MAPK14 function in governing VSMC phenotype. Further support for the above findings was observed in quantitative reverse transcription polymerase chain reaction (qRT‒PCR), which revealed a significant increase in *Srf* and a decrease in *Runx1* and *Runx2* mRNA in the aortas of the KO mice (Fig. 4d). The upregulation of SRF and downregulation of RUNX2 were further validated by Western blotting (Fig. 4e, f). Interestingly, although the motif activity of the MEF2 family is closely associated with VSMC differentiation and is upregulated in the KOs, the protein levels of MEF2D and MEF2C were not changed in the KOs (Fig. 4e, f). These results collectively indicate that MAPK14 reciprocally modulates the key TFs SRF and RUNX1/2 to control VSMC phenotypic alterations during AAA formation.

### Integration of snATAC-seq, ChIP-seq, and bulk RNA-seq identifies MYOCD/SRF/CArG-dependent transactivation of the contractile gene program caused by VSMC loss of MAPK14

**Fig. 5:**
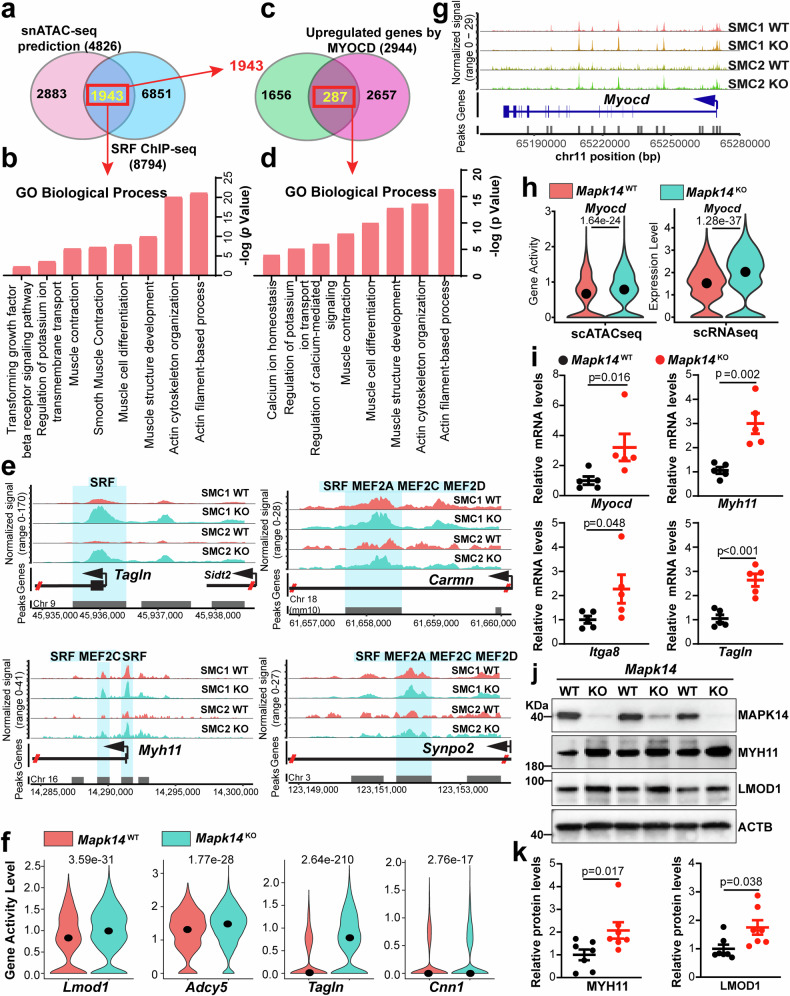
Integration of snATAC-seq, ChIP-seq, and bulk RNA-seq identifies MYOCD/SRF/CArG-dependent transactivation of the contractile gene program caused by VSMC loss of MAPK14. **a** Venn diagram showing overlapping SRF target genes based on both snATAC-seq of the *Mapk14*^WT^ and *Mapk14*^KO^ datasets and SRF ChIP-seq of human coronary artery smooth muscle cells (HCASMCs). **b** GO biological process analysis of the overlapping genes identified in (**a**). **c** Venn diagram showing the 1943 overlapping genes from (**a**) and genes upregulated by MYOCD overexpression in HCASMCs based on bulk RNA-seq. **d** GO biological process analysis of the overlapping genes identified in (**c**). **e** Chromatin accessibility of the indicated TFs in the indicated contractile gene locus in SMC clusters 1 and 2. **f** Violin plots showing the gene activity of the representative contractile genes in total VSMCs from *Mapk14*^WT^ and *Mapk14*^KO^ mice. **g** Chromatin accessibility at the *Myocd* gene locus in SMC clusters 1 and 2 in AAs from *Mapk14*^WT^ and *Mapk14*^KO^ mice. **h** Violin plots showing the gene activity (left) and gene expression (right) of *Myocd* in total VSMCs from *Mapk14*^WT^ versus *Mapk14*^KO^ mice. **i** qRT‒PCR analysis of *Myocd* and the indicated contractile genes (*Myh11*, *Itga8*, *Tagln)* in AAs from *Mapk14*^WT^ and *Mapk14*^KO^ mice infused with Ang II for 7 days (*n* = 5 per group). **j** Representative Western blot images of the indicated contractile proteins (MYH11 and LMOD1) in AAs from *Mapk14*^WT^ and *Mapk14*^KO^ mice infused with Ang II for 7 days. **k** Quantification of MYH11 and LMOD1 protein levels (*n* = 7 per group). The data were analyzed via Student’s *t* tests for (**i**, **k**)

Data above suggests that SRF may serve as a major transcriptional effector of MAPK14 to control VSMC differentiation. We next examined the motif coverage of SRF predicted by Signac. We prioritized genes with SRF peaks residing in proximal promoter regions, defined as those within −3 kb from the transcription start site of the target genes across the genome, which resulted in 4826 genes in the mouse genome. On the other hand, we conducted SRF ChIP-seq in cultured human coronary artery smooth muscle cells (HCASMCs), which revealed 8794 genes harboring SRF peaks throughout the human genome. Although HCASMCs are not derived from aortas, SRF-mediated transcriptional regulation is broadly conserved across vascular beds. Therefore, this dataset provides a valuable framework for identifying direct SRF target genes dysregulated in AAA. Cross-referencing these two datasets yielded 1943 genes, which are potential SRF/CArG targets conserved in both humans and mice. Pathway analysis of these targets showed a strong enrichment in actin filament-based processes, muscle contraction and differentiation (Fig. 5a, b), which was consistent with the enriched pathways in the VSMCs from the KO mice demonstrated by snRNA-seq, as shown in Fig. 2. To more precisely define the extent to which these target genes are regulated by the MYOCD/SRF/CArG triad, we further cross-referenced these 1943 genes with our MYOCD bulk RNA-seq data.^8^ This analysis revealed 287 target genes whose expression was induced by MYOCD (>2-fold). GO analysis further demonstrated strong enrichment in muscle development, muscle contraction, and the relevant ion channel signaling pathways (Fig. 5c, d). Because SMC1 and SMC2 are the major cell clusters with high scores of contractile gene activity, we next focused on the assessment of MYOCD/SRF/CArG target genes in these two clusters. As expected, Signac predicted known VSMC contractile and contractile-associated genes, such as *Tagln*, *Myh11*, *Carmn*, and *Synpo2*. VSMC contractile genes are downregulated in an Ang II-induced AAA model.^47^ These target genes possess increased chromatin accessibility in the regions encompassing CArG elements in the KO compared with the WT mice (Fig. 5e). Interestingly, *Carmn, Myh11, and Synpo2* exhibited increased chromatin accessibility in regulatory regions harboring both SRF and MEF2 motifs in KOs, suggesting that SRF and MEF2 cooperate in the regulation of VSMC contractile gene transcription by MAPK14 (Fig. 5e). Consistently, the gene activity levels of the VSMC contractile-associated genes, including *Lmod1*, *Adcy5*, *Tagln*, and *Cnn1*, were significantly elevated in the KO mice, as revealed by snATAC-seq (Fig. 5f). The specificity and transcriptional activity of SRF require the cofactor MYOCD.^48^ We thus assessed the chromatin accessibility profile at the *Myocd* gene locus. Interestingly, multiple peaks within the *Myocd* gene locus exhibited increased chromatin accessibility in KOs (Fig. 5g). These findings indicate that loss of MAPK14 enables a more active chromatin status for *Myocd* transcription. Consistently, increased *Myocd* gene activity and expression were revealed by sn-ATAC-seq and RNA-seq, respectively in KO relative to WT (Fig. 5h). qRT-PCR analysis showed significantly increased *Myocd* and downstream target contractile genes, such as *Myh11*, *Itga8*, and *Tagln*, in the aortas of the KO mice (Fig. 5i). Increased contractile marker expression in KOs was further confirmed by Western blotting (Fig. 5j, k). Interestingly, saline-infused WT and KO AAs displayed comparable levels of SMC contractile markers (Supplementary Fig. 8a, b), suggesting that the influence of MAPK14 loss on SMC contractile gene expression is AAA-context dependent. Taken together, these data strongly support that loss of MAPK14 enhances the transcriptional activity of the MYOCD/SRF/CArG triad, thereby promoting the contractile gene program in VSMCs during AAA formation.

### *BCL2* is a novel target of the MYOCD/SRF/CArG triad upregulated by MAPK14 loss in VSMCs

**Fig. 6:**
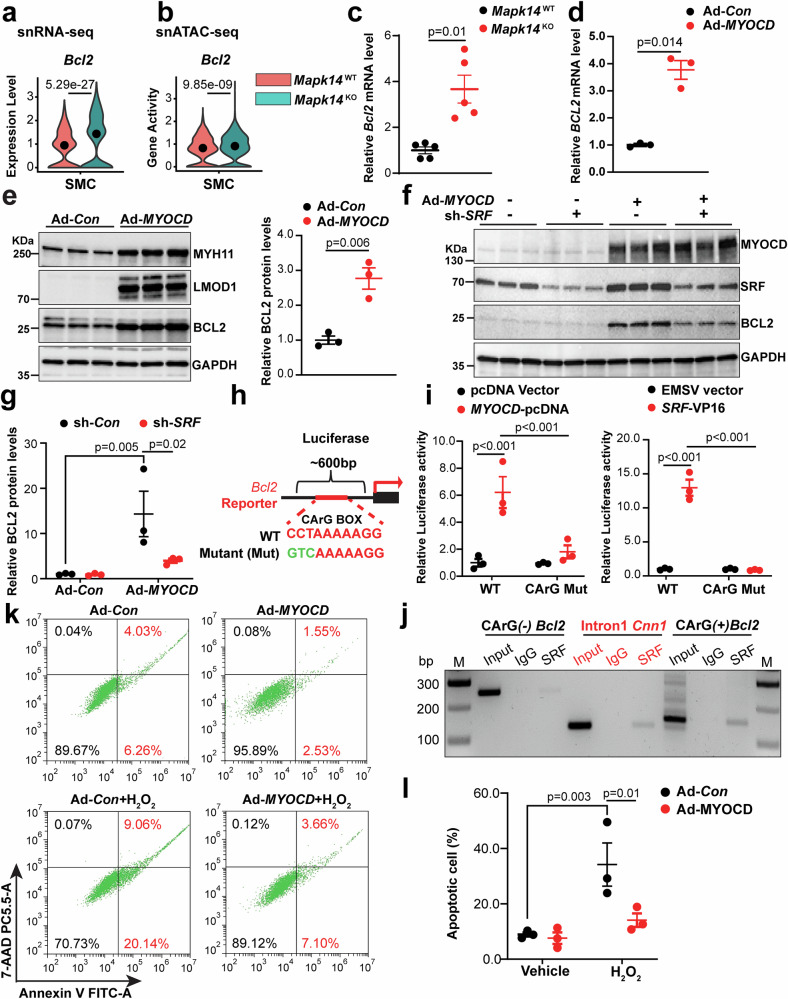
*Bcl2* is a novel target of MYOCD/SRF/CArG triad upregulated by VSMC loss of MAPK14. Violin plots showing the gene expression (**a**) and gene activity (**b**) of *Bcl2* based on snRNA/ATAC-seq in *Mapk14*^WT^ and *Mapk14*^KO^ mice. **c** qRT‒PCR validation of *Bcl2* gene expression in AAs from *Mapk14*^WT^ and *Mapk14*^KO^ mice infused with Ang II for 7 days (*n* = 5 per group). **d** qRT‒PCR analysis of *Bcl2* gene expression in human aortic smooth muscle cells (HASMCs) transduced with Ad-*MYOCD* and empty control virus (Ad-Con) (*n* = 3). **e** Western blotting of the indicated proteins in HASMCs transduced with Ad-*MYOCD* or Ad-Con and quantification of BCL2 protein levels (*n* = 3). **f**, **g** HASMCs were transduced with Ad-*MYOCD* or Ad-Con followed by transduction with Ad-shSRF or Ad-shCon prior to Western blotting for the indicated proteins and quantification of BCL2 protein levels under the indicated conditions. **h** Schematic of the luciferase reporter of the mouse *Bcl2* (−645 bp) promoter encompassing the conserved CArG box and the mutagenesis strategy for this CArG box. **i** Relative luciferase activity of the WT and CArG mutant *Bcl2* reporters induced by MYOCD or SRF in PAC1 SMCs. The data were normalized to the Renilla internal control and are presented as fold changes relative to the control groups (*n* = 3). **j** Semi-quantitative PCR for SRF chromatin immunoprecipitation (ChIP) in the mouse VSMC cell line MOVAS. Note: SRF is enriched in the CArG-containing region of the proximal promoter of *Bcl2*. An intronic *Cnn1* region flanking an established functional CArG box and a 3’UTR of *Bcl2* without a CArG box served as the positive and negative controls, respectively. **k**, **l** Flow cytometry for Annexin V and 7-AAD staining was used to assess H_2_O_2_-induced apoptosis in HASMCs ± Ad-MYOCD cells, and the percentage of apoptotic cells was quantified (*n* = 3). The data were analyzed via Student’s *t* tests for (**c,** **d**, **e**) and via two-way ANOVA followed by the Bonferroni post hoc correction for (**g,** **i**, **l**)

In addition to the known contractile genes that were upregulated in KOs revealed by snRNA/ATAC-seq, we also discovered a number of novel genes predicted as SRF transcriptional targets. One such gene is *Bcl2*, which is known to inhibit VSMC apoptosis during AAA formation.^49^ Because reduced VSMC apoptosis triggered by Ang II was observed in aortas from MAPK14 KO mice (Fig. 1g–i), we asked whether VSMC-MAPK14 influences *Bcl2* gene expression and thus apoptosis. Indeed, both the gene expression and gene activity of *Bcl2* were significantly increased in KO VSMCs, as revealed by snRNA/ATAC-seq (Fig. 6a, b). The induction of *Bcl2* in VSMCs from KO mice was validated by qRT‒PCR using total RNA isolated from the aortas of the mice infused with Ang II for 7 days (Fig. 6c). To determine whether *Bcl2* is a novel transcriptional target of the MYOCD/SRF/CArG triad, we first determined whether MYOCD can induce *Bcl2* gene expression. Indeed, compared with control HCASMCs, HCASMCs transduced with Ad-MYOCD showed much higher levels of *BCL2* mRNA (Fig. 6d). Furthermore, Western blot analysis revealed that overexpression of MYOCD dramatically increased BCL2 protein expression; these increases were partially reversed by short hairpin RNA (shRNA)-mediated *SRF* (sh*SRF*) knockdown in HCASMCs (Fig. 6e–g). These results suggest that *BCL2* is a new transcriptional target of MYOCD/SRF. Consistent with this notion, our CArGome data predicted a consensus and conserved CArG element in the proximal promoter of the *Bcl2* gene locus (Fig. 6h). To test the functionality of this CArG box, we PCR-cloned the −645 bp *Bcl2* mouse promoter encompassing this predicted CArG box into a luciferase reporter and tested the luciferase activity in PAC1 SMCs.^50^ Both MYOCD and SRF markedly activated *Bcl2* luciferase reporter activity, and this activation was abolished by a mutation in this predicted CArG box (Fig. 6i). Furthermore, similar to the intronic *Cnn1* CArG element reported previously,^51,52^ the SRF ChIP assay revealed enriched SRF binding to the CArG-containing region within the *Bcl2* proximal promoter but not to the 3-UTR of *Bcl2* lacking a predicted CArG element (Fig. 6j). Finally, the overexpression of MYOCD protected VSMCs from H_2_O_2_-induced apoptosis, as revealed by flow cytometry with Annexin V and 7-aminoactinomycin D (7-AAD) labeling (Fig. 6k, l). To extend these findings to human disease, immunohistochemistry revealed significantly lower BCL2 protein levels in the medial layer of AAA tissues compared with non-AAA aortas, and an immuno-RNA fluorescence in situ hybridization assay further revealed decreased *MYOCD* mRNA levels in AAA tissues (Supplementary Fig. 9). These data demonstrate that in addition to the classic contractile genes, the loss of MAPK14 triggers *Bcl2* gene transcription via the MYOCD/SRF/CArG pathway, which likely contributes to the reduced apoptosis observed in MAPK14 KO mice.

### MAPK14 loss in VSMCs downregulates the gene expression of degenerative genes and promotes MRTFA ubiquitin proteasome degradation

**Fig. 7:**
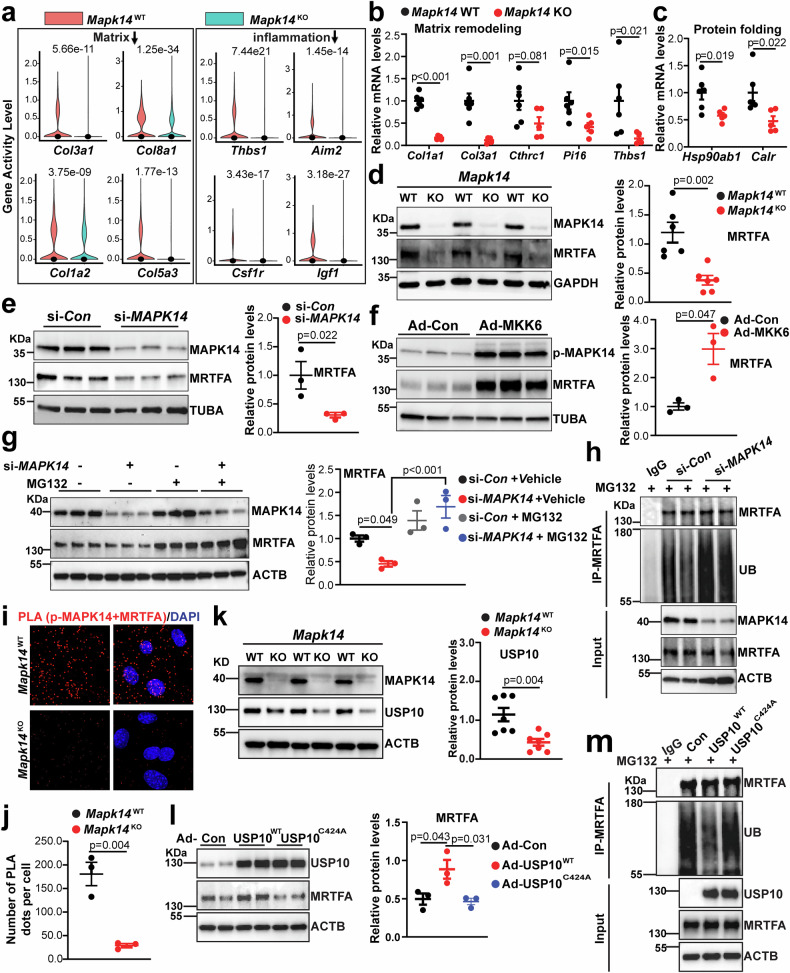
MAPK14 loss in VSMCs downregulates the expression of degenerative genes and promotes MRTFA ubiquitin proteasome degradation. **a** Violin plot depicting the gene activity of matrix- and inflammation-related genes in AAs from *Mapk14*^WT^ and *Mapk14*^KO^ mice. **b, c** qRT‒PCR validation of matrix genes (*Col1a1*, *Col3a1*, *Cthrc1*, *Pi16*, *Thbs1*) and unfolded protein response (UPR) chaperone genes (*Hsp90ab1* and *Calr)* in *Mapk14*^WT^ and *Mapk14*^KO^ mice infused with Ang II for 7 days (*n* = 5‒6 per group). **d** Representative Western blot images of the indicated proteins in *Mapk14*^WT^ and *Mapk14*^KO^ mice infused with Ang II for 7 days (left) and quantification of MRTFA protein levels (*n* = 6 per group) (right). **e** Representative Western blot images of the indicated proteins in HASMCs ± si-*MAPK14* (right) and quantification of MRTFA protein levels (*n* = 3) (left). **f** HASMCs were transduced with Ad-HA-MRTFA for 24 h, followed by transduction with equal amounts of Ad-Empty or Ad-MKK6 (MOI = 50) for 48 h. Representative Western blot images of the indicated proteins (left) and quantification of MRTFA protein are shown (*n* = 3) (right). **g** HASMCs were transfected with an equal amount of siRNA control (si-*Con*) or siRNA specific for MAPK14 (si-*MAPK14*) for 48 h, followed by treatment with MG132 (10 μM) for 8 h prior to protein extraction. Representative Western blot images of the indicated proteins (left) and quantification of the MRTFA protein are shown (*n* = 3) (right). **h** HASMCs were transfected with equal amounts of si-*Con* or si-*MAPK14* followed by MG132 (10 µM) treatment for 8 h before protein extraction for immunoprecipitation of MRTFA, followed by Western blotting for the indicated proteins. A total of 1/100 of the total cell lysates were used as input controls, and representative Western blot images for the indicated proteins are shown (*n* = 3). **i**, **j** Proximity ligation assay (PLA) for MRTFA and pMAPK14 in mouse aortic SMCs (MASMCs) isolated from *Mapk14*^WT^ and *Mapk14*^KO^ mice and quantification of the number of PLA dots per cell (*n* = 3). **k** Representative Western blot images of the indicated proteins in *Mapk14*^WT^ and *Mapk14*^KO^ mice infused with Ang II for 7 days (right) and quantification of USP10 protein levels (*n* = 7 per group) (left). **l** HASMCs were transduced with Ad-USP10^WT^ or Ad-USP10^C424A^ for overexpression of WT USP10 or the catalytically inactive mutant USP10 (loss of deubiquitination activity), respectively, or with equal amounts of Ad-Empty as a control for 36 h; representative Western blot images of the indicated proteins (left) and the quantification of MRTFA protein are shown (*n* = 3) (right). **m** HASMCs were transduced with equal amounts of Ad-Con, Ad-USP10^WT^, or Ad-USP10^C424A^ for 36 h, followed by treatment with MG132 (10 μM) for 8 h prior to protein extraction for immunoprecipitation of MRTFA and Western blotting for the indicated proteins. A total of 1/100 of the total cell lysates were used as input controls, and representative Western blot images for the indicated proteins are shown (*n* = 3). The data were analyzed via Student’s *t* test in (**b**, **c**, **d**, **e**, **f**, **j**, **k**); two-way ANOVA followed by the Bonferroni post hoc correction for (**g**); and one-way ANOVA followed by the Tukey post hoc test for (**l**)

SnATAC-seq revealed significant downregulation of degenerative genes, including matrix and fibrotic genes (*Col3a1*, *Col8a1*, *Col1a2*, and *Col5a3*), and inflammation and cell death genes (*Thbs1*, *Aim2*, *Csf1r*, and *Igf1*), in KO mice (Fig. 7a). Consistently, qRT‒PCR analysis of AAs from the mice infused with Ang II for 7 days showed reduced gene expression of fibrotic and inflammatory genes (*Col1a1*, *Col3a1*, *Cthrc1*, *Pi16*, and *Thbs1*) in KO AAs (Fig. 7b). Consistent with the reduced protein levels of ER stress/chaperone markers in KOs (Fig. 1g), qRT-PCR showed attenuation of *Hsp90ab1* and *Calr* gene expression in KOs (Fig. 7c). We previously reported that MRTFA promotes aortic dissection and aneurysm formation via MAPK14.^19^ MRTFA has been documented as a critical regulator that promotes vascular fibrosis, inflammation, and cell death, contributing to vascular disease.^19,20,53^ We thus asked whether MAPK14, in turn, activates MRTFA in a feedforward loop that amplifies VSMC degeneration. Surprisingly, although no significant difference in MRTFA protein abundance was detected in the WT and KO aortas at the baseline level (Supplementary Fig. 10a), the MRTFA protein was nearly abolished in the aortas of the KO mice compared with those of the WT mice infused with Ang II for 7 days (Fig. 7d). Consistently, siRNA-mediated *MAPK14* knockdown in human aortic smooth muscle cells (HASMCs) significantly decreased the MRTFA protein level (Fig. 7e). Conversely, activation of MAPK14 via forced expression of the constitutively active MAPK14 upstream kinase MKK6 increased the level of exogenous HA-tagged MRTFA protein in HASMCs (Fig. 7f).^26^ To determine whether the observed protein alterations originated from changes at the mRNA level, we assessed the influence of MAPK14 on *Mrtfa* gene expression under different conditions. Interestingly, snRNA-seq revealed a slight increase in *Mrtfa* gene expression in the KO VSMCs compared with the WT VSMCs (Supplementary Fig. 10b). However, the mRNA levels of endogenous *MRTFA* were not changed upon either siRNA-mediated *MAPK14* gene knockdown or MAPK14 activation by MKK6 overexpression in HASMCs (Supplementary Fig. 10c, d). These findings indicate that the striking influence of MAPK14 on the MRTFA protein is not due to its mRNA alterations. To determine whether MAPK14 influences MRTFA protein proteasome degradation, we depleted *MAPK14* in HASMCs with siRNA followed by treatment with the proteasome inhibitor MG132. MG132 completely rescued the downregulation of the MRTFA protein caused by *MAPK14* knockdown (Fig. 7g). Furthermore, immunoprecipitation (IP) assays showed that si*MAPK14*-treated HASMCs displayed elevated levels of the ubiquitinated form of MRTFA (Fig. 7h), supporting a previously unknown function of MAPK14 in preventing MRTFA proteasome degradation. To determine whether the influence of MAPK14 on MRTFA protein stability requires their physical interaction, we performed proximity ligation assay (PLA), a well-accepted approach for determining the physical interaction between proteins.^54,55^ While abundant MAPK14 and MRTFA PLA signals were observed in both the cytosol and nucleus of WT mouse aortic SMCs (MASMCs), these signals were virtually abolished in KO cells (Fig. 7i, j), supporting the physical interaction between MAPK14 and MRTFA in WT VSMCs. Interestingly, USP10, a deubiquitinase previously reported to interact with and stabilize the MRTFA protein,^56^ was significantly reduced in KO AAs (Fig. 7k), suggesting that USP10 may serve as a critical mediator by which MAPK14 stabilizes the MRTFA protein. Indeed, adenovirus-mediated overexpression of WT USP10 (Ad-USP10^WT^), but not a catalytically inactive USP10 mutant (Ad-USP10^C424A^), significantly increased the MRTFA protein level (Fig. 7l). This finding is consistent with the reduced MRTFA ubiquitination observed in Ad-USP10^WT^-transduced HASMCs compared with that of the Ad-USP10^C424A^ mutant, as revealed by co-IP (Fig. 7m). Collectively, these results reveal a novel mechanism in which MAPK14 stabilizes the MRTFA protein through USP10-mediated protection from proteasomal degradation, thereby reinforcing a feed-forward pathway by which MAPK14 and MRTFA cooperate to drive VSMC degeneration and AAA formation.

## Discussion

**Fig. 8:**
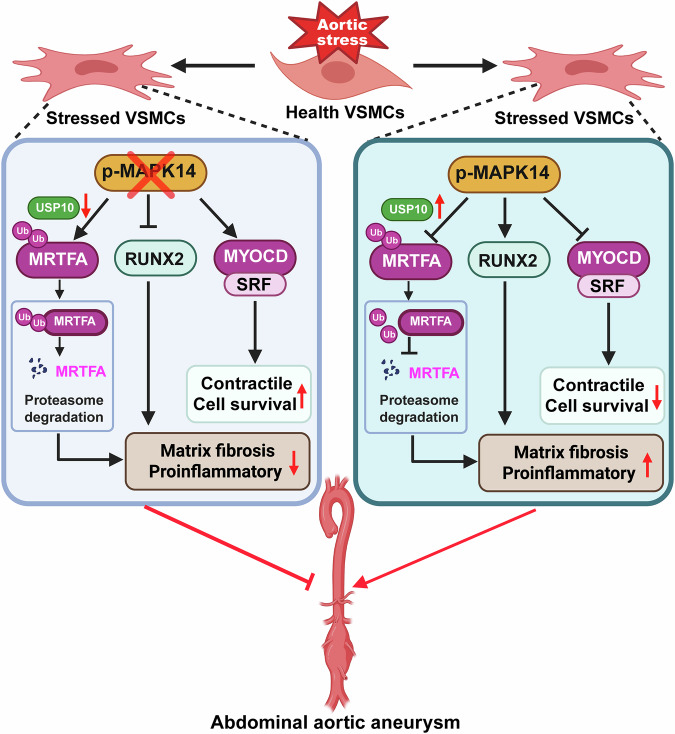
The graphical abstract of this study (created with https://www.biorender.com/). Aortic stress activates MAPK14 in VSMCs, which suppresses the MYOCD/SRF/CArG triad-dependent transactivation of contractile and pro-survival genes and inhibits the ubiquitination and proteasomal degradation of MRTFA while increasing RUNX2 expression. These coordinated transcriptional and posttranslational events diminish VSMC contractility and survival, increase fibrosis and inflammation, and collectively drive VSMC degeneration and abdominal aortic aneurysm (AAA) formation

The present study integrated snRNA/ATAC-seq, ChIP-seq, and extensive wet lab validation to reveal how MAPK14 regulates key transcriptional networks that drive VSMC phenotypic degeneration and AAA formation. We showed that VSMC loss of MAPK14 protected against Ang II-induced VSMC degeneration, resulting in reduced ER stress, apoptosis, and fibrosis, along with an expansion of the contractile VSMC population and a reduction in the number of inflammatory and fibrotic SMC clusters. Multi-omics integration revealed the MYOCD/SRF/CArG triad as a key downstream transcriptional effector of MAPK14 loss to activate contractile genes and BCL2, a novel transcriptional target that likely contributes to VSMC survival. In contrast, MAPK14 loss diminished the expression of the MRTFA protein, a key cofactor that transactivates proinflammatory and fibrotic gene programs. Mechanistically, we demonstrated that MAPK14 stabilizes the MRTFA protein by maintaining USP10, which prevents the ubiquitination and proteasomal degradation of MRTFA. Collectively, these findings demonstrate how MAPK14 orchestrates the primary transcriptional machinery to drive VSMC degeneration and promote AAA formation (Fig. 8).

The contractile to proinflammatory, fibrotic, and osteochondrogenic degenerative VSMC phenotypic transitions have long been recognized as major contributors to aortic aneurysms.^57–59^ The advent of scRNA-seq technology coupled with VSMC lineage tracing has enabled more precise characterization of such phenotypic alterations, further reinforcing their role in aneurysm pathogenesis.^42,60^ Using these approaches, a recent study from Shen’s laboratory elegantly demonstrated the highly dynamic and remarkable transdifferentiation of VSMCs in an Ang II-induced aortic aneurysm model.^42^ While the precise contribution of these transdifferentiated VSMCs to aortic aneurysm progression remains to be fully elucidated, particularly through sophisticated genetic tools targeting specific VSMC cluster(s), the proinflammatory, fibrotic, death-prone features of those VSMC clusters suggest their involvement in VSMC degeneration and aortic aneurysm. Our snRNA-seq and snATAC-seq data consistently revealed contractile, EC-SMC, and fibroblast-like SMC clusters in an Ang II-induced AAA model. Notably, both assays revealed a significant increase in the contractile VSMC population and a reduction in immune cells and fibroblasts upon MAPK14 loss. These results align with the phenotype demonstrated by our wet lab experiments, indicating a crucial role of SMC-MAPK14 in aortic destruction and AAA formation. The overrepresentation of immune and fibroblast populations in WT aortas revealed by snRNA-seq and ATAC-seq corresponds with increased fibrosis, oxidative stress, cell proliferation, and proinflammatory cell infiltration in the adventitia of WT mice, as validated by wet lab assays. Because MAPK14 depletion reduces the secretion of proinflammatory mediators such as IL6 and MRTFA loss blunts MAPK14 activation and downstream proinflammatory gene expression,^19^ we propose that a paracrine pathway governed by the MAPK14–MRTFA feedback loop in degenerative VSMCs mediates adventitial remodeling.

A key paradigm emerging from our current study is the integration of sn-ATAC-seq, ChIP-seq, and bulk RNA-seq analyses to collectively pinpoint the MYOCD/SRF/CArG regulatory axis as a primary transcriptional effector of MAPK14 loss to maintain VSMC differentiation. Specifically, cross-referencing snRNA-seq and SRF-ChIP-seq revealed 1943 SRF/CArG target genes enriched in the SMC contraction and ion channel pathways, with 287 of these target genes induced by the MYOCD transcriptional cofactor. Further examination of contractile or contractile-associated target genes revealed that MAPK14 loss resulted in increased chromatin accessibility in CArG-containing regions located in intronic or promoter regions. These lines of evidence strongly support the role of MYOCD/SRF/CArG as a major transcriptional effector of MAPK14 in VSMCs during AAA formation. It should be noted, chromatin accessibility only indicates the potential for TF recruitment rather than active transcription, as the latter depends on the availability of sufficient levels of TFs.^61^ In line with these findings, our wet lab studies showed that both SRF and MYOCD were significantly increased in MAPK14 KOs, further underscoring their potential roles in mediating MAPK14 function in VSMCs. Additionally, our bioinformatic analysis revealed a strong association of the MEF2 TF family with the VSMC contractile gene program, which is consistent with recent publications.^41,42^ However, Signac predictions indicated that only a handful of contractile genes possessed open chromatin peaks encompassing both MEF2 and SRF motifs in close proximity, suggesting that SRF and MEF2 are unlikely to cooperatively transactivate VSMC differentiation. Nevertheless, the definitive role of MEF2 in VSMC differentiation requires thorough transcription assays and genetic MEF2 family knockout (KO) mouse models. In addition, our ATAC-seq analysis identified several novel TFs, including RUNX2, ELF1, and GATA3, that are associated with VSMC degeneration. Preliminary siRNA-mediated depletion of RUNX2 and ELF1 in HASMCs reduced the expression of select inflammatory and extracellular matrix-remodeling genes, whereas contractile gene expression remained unaffected (Supplementary Fig. 11). These findings provide initial functional support for their involvement in VSMC phenotypic modulation; however, their definitive pathophysiological roles need to be established using genetic models in relevant disease settings.

The reduction in the MRTFA protein in MAPK14 KOs is profound, revealing a previously unknown role of MAPK14 in regulating MRTFA protein stability. This finding, along with the observed reduction in MAPK14 activity in MRTFA-deficient VSMC cells, supports a novel positive feedback loop between MRTFA and MAPK14 in driving VSMC degeneration and AAA formation.^19^ Through a series of in vitro experiments, we demonstrated that MAPK14 stabilizes MRTFA by preventing ubiquitin proteasome degradation mediated by USP10, an understudied deubiquitinase in VSMCs. These findings, together with our recent report on a similar role of the long noncoding RNA *INKILN*, underscore that MRTFA protein stability is modulated by both coding and noncoding pathways, adding a new regulatory layer beyond its well-established cytonuclear translocation dynamics.^56^ Interestingly, despite the nearly complete loss of the MRTFA protein in MAPK14 KOs, *Myocd* expression is significantly increased, which is consistent with the increased chromatin accessibility at the *Myocd* gene locus revealed by snATAC-seq. These findings suggest that MAPK14 loss may, at least in part, enable epigenetic activation of *Myocd* transcription. The precise mechanisms underlying this process, however, require further investigation. These findings support a unique bidirectional regulatory effect of MAPK14 on *Myocd* mRNA expression and MRTFA protein stability to collectively influence the pathogenesis of aortic aneurysms given their opposing functional consequences in vascular pathology.

In addition to MRTFA/MYOCD, we identified a positive association of RUNX family members with VSMC degeneration. RUNX2 was originally discovered as a master regulator of VSMC calcification.^62–64^ Recent studies have expanded this original pro-osteogenesis function to proinflammation, fibrosis, pro-osteoclast, as well as VSMC phenotypic switching.^43,65^ The positive association of RUNX2 with degenerative VSMC clusters observed in our study is consistent with its well-established role in vascular pathology. A previous study reported that activated MAPK14 enhances RUNX2 transcriptional activity and promotes vascular calcification in cultured VSMCs. Our findings of reduced motif activity and expression of RUNX2 in VSMCs from MAPK14 KO mice further support the regulatory role of MAPK14 in RUNX2 transactivation of downstream genes and, consequently, vascular calcification.^66^ Given the causative role of vascular calcification in aortic aneurysm progression,^67–69^ it will be essential to investigate whether MAPK14 loss in VSMCs reduces aortic calcification in vivo, providing further insight into the MAPK14/RUNX2 axis as a therapeutic target for aortic aneurysm.

Several limitations should be acknowledged in our current study. First, the *Sm22-*Cre driver used in this study has intrinsic activity in myofibroblasts; thus, potential confounding effects on the VSMC phenotype should be acknowledged. Second, although our integrative multi-omics approach captures the altered regulation of the SRF/MYOCD/CArG-mediated transcriptional program of VSMC contractile genes, direct assays for SRF transcriptional activity under MAPK14 deficiency were not performed. We recognize this as a limitation and note it as a future direction. Third, although we delineated the mechanism by which MAPK14 regulates MRTFA protein stability, the specific TF(s) that cooperate with MRTFA to drive VSMC degeneration remain to be defined.

In summary, through the integration of single-nucleus multi-omics, SRF ChIP-seq, and rigorous wet lab validation, we comprehensively delineated the major transcriptional effectors regulated by MAPK14, a key upstream signaling molecule that facilitates VSMC degeneration and aneurysm formation. Our findings reveal that MAPK14 governs the reciprocal regulation of the MYOCD/SRF and MRTFA/RUNX TFs, highlighting their distinct yet interconnected roles in AAA pathogenesis. These results not only elucidate the complex molecular mechanisms underlying the protective effect of MAPK14 loss in VSMCs during AAA formation but also provide novel insights into the bidirectional regulation of MYOCD and MRTFA by MAPK14. Collectively, these findings suggest that targeting the VSMC-MAPK14 axis may represent a promising therapeutic strategy for mitigating AAA progression.

## Materials and methods

### Experimental animals

All animal procedures were approved by the Institutional Animal Care and Use Committee at Augusta University. Mice were maintained in temperature-controlled rooms with a 12/12-h light/dark cycle. VSMC-specific *Mapk14* KO mice were generated by crossing *Mapk14*^f/f^ mice with *Sm22*-Cre^+/-^ transgenic mice as previously reported.^19^ These mice were further bred with *Apoe*^-/-^ mice to generate experimental VSMC-*Mapk14* KO (*Sm22*-Cre^+/-^/*Mapk14*^f/f^/*Apoe*^-/-^) mice. Littermate control *Mapk14*^f/f^/*Apoe*^-/-^ mice were used as WT controls in the following experiments given that no discernable difference in aneurysm phenotype was observed between *Mapk14*^f/f^/*Apoe*^-/-^ and *Sm22*-Cre^+/-^/*Mapk14*^+/+^/*Apoe*^-/-^ mice.^19^ All the mice were on the C57BL/6J background and were born at the expected Mendelian ratio. Sixteen- to eighteen-week-old mice were challenged with angiotensin II (Ang II, Cat# H-1706; Bachem) at a dose of 1000 ng/kg/min or saline through subcutaneous mini-pumps (Alzet, model 2004) for 1 week. AAs were then isolated for wet lab or snRNA-seq and snATAC-seq experiments.

### VSMC culture and treatment

Human aortic smooth muscle cells (HASMCs) (Lonza, CC-2571) and human coronary artery smooth muscle cells (HCASMCs) (ATCC, PCS-100-021) were cultured in VascuLife® SMC Medium (Lifeline Cell Technology, ll0014) at 37 °C with 5% CO_2_ in a humidified incubator. MOVAS (mouse primary vascular aortic smooth muscle cells) (ATCC, CRL-2729) were cultured in Dulbecco’s modified Eagle’s medium (DMEM) (Gibco, 11320-033) supplemented with 0.2 mg/ml G418 and 10% fetal bovine serum (FBS) (Cytiva, SH30071.03). PAC1 SMCs (Cellosaurus, CVCL-U511) were cultured in Dulbecco’s modified Eagle’s medium (DMEM) (Gibco, 11320-033) supplemented with 1% penicillin‒streptomycin (Sigma‒Aldrich, P4333) and 10% FBS. MASMCs were prepared and maintained as previously described.^70^ Only the first eight passages of human VSMCs and three passages of primary MASMCs were used for all the experiments. For siRNA transduction, HASMCs were transfected with ON-TARGETplus human *MAPK14* siRNA pool (Horizon, L-003512-00-0010) and ON-TARGETplus Nontargeting Control Pool (Horizon, D-001810-10-20) at 70–80% confluence using PepMute ^TM^ Plus siRNA transfection reagent according to the instructions (SignaGen Laboratories, SL100571). After 48 h of transfection, cells were serum-starved overnight and subsequently treated with 10 μM MG132 (Cell Signaling Technology, 2194) for 8 h before protein extraction for immunoprecipitation and Western blotting. The transduction of different adenoviruses into HASMCs was conducted as described previously.^26^ Information about Ad-MKK6, Ad-MYOCD, Ad-shSRF and the empty control adenovirus is provided elsewhere.^8,26,56^ Adenoviruses carrying cDNA encoding WT human USP10 (Ad-USP10), the catalytically inactive USP10 mutant Ad-USP10^C424A^, and the corresponding control adenovirus were generated by VectorBuilder, Inc.

### Histological measurement, immunofluorescence microscopy, and ROS detection

Paraffin-embedded AA cross sections (5 µm) were deparaffinized, rehydrated, and then subjected to standard hematoxylin and eosin (H&E), Masson’s trichrome (StatLab, KTMTR2), or Picrosirius Red (Abcam, ab150681) staining for morphometric analysis. The collagen content was quantified as the percentage of positively stained area via ImageJ software (version 1.54i 03). Immunohistochemistry (IHC) DAB staining for pMAPK14 was performed using a Mouse and Rabbit Specific HRP/DAB IHC Detection Kit (Abcam, ab236466). After IHC staining, the sections were counterstained with hematoxylin, dehydrated and mounted with mounting medium (Thermo Scientific, 8312-4). The staining signals were examined using an Olympus BX43 microscope.

For immunofluorescence staining, the above deparaffinized, rehydrated sections were subjected to antigen retrieval using citrate solution (pH = 9) (Dako, S236784-2). The sections were blocked in blocking solution (Dako, X0909) for 30 min at room temperature (RT) and incubated with primary antibodies overnight at 4 °C. After being washed with phosphate-buffered saline (PBS), the sections were incubated with secondary antibodies for 1 h at RT. Finally, the sections were washed with PBS and mounted with ProLong™ Glass Antifade Mountant containing NucBlue™ (Thermo Scientific, P36918). Immunostained images were acquired via confocal microscopy (LSM 900, Zeiss). Information on the primary and secondary antibodies is provided in Supplementary Table 1.

Dihydroethidium (DHE) (Thermo Scientific, D23107) was used to detect the ROS content in AAs as previously described.^71^ Briefly, frozen sections were washed with PBS and then incubated with 10 µM DHE for 30 min at 37 °C in the dark. The ROS signal was acquired via confocal microscopy (LSM 900, Zeiss) and quantified with ImageJ.

### TUNEL and Annexin V/7-AAD staining

TUNEL staining was performed on paraffin-embedded sections. Briefly, the sections were deparaffinized, rehydrated, and subjected to antigen retrieval using citrate solution at pH = 6 (Dako, S236984-2). TUNEL labeling was performed via an In Situ cell death detection kit (Roche, 11684795910) according to the manufacturer’s instructions. Fluorescence images were acquired using a confocal microscope (LSM 900, Zeiss). Annexin V/7-AAD was applied to assess cell death in cultured cells by flow cytometry. FITC Annexin V (an apoptotic cell marker) and 7-ADD (a necrotic marker) were applied using the FITC Annexin V Apoptosis Detection Kit (BioLegend, 640922) following the manufacturer’s instructions. H_2_O_2_-treated HASMCs were harvested, washed with cold cell staining buffer (BioLegend, 420201), and resuspended in Annexin V binding buffer at a density of 0.5 × 10^7^ cells/ml. The cells were stained with FITC‒Annexin V and 7-AAD for 15 min at 25 °C in the dark, followed by incubation with Annexin V binding buffer. The samples were immediately analyzed using a flow cytometer (Novocyte Quanteon, BD Biosciences), and the data were analyzed via FlowJo software (BD Biosciences).

### Enzyme-linked immunosorbent assay (ELISA)

After 48 h of transfection with either a human MAPK14 siRNA pool or a nontargeting control pool, HASMCs were serum-starved overnight and subsequently treated with 20 ng/ml TNFα (R&D Systems, 210-TA-020) for 24 h. The supernatant from the culture medium was collected to measure secreted IL-6 via a human IL-6 ELISA Kit per the manufacturer’s instructions (Thermo Scientific, EH2IL6).

### Western blot and immunoprecipitation for the ubiquitination assay

Homogenized aortic tissue and cultured HASMCs were lysed in RIPA buffer (Thermo Scientific, 89900) containing protease and phosphatase inhibitors (Thermo Scientific, 78422) for total protein extraction. Equal amounts of protein (15 μg/lane) were subjected to SDS polyacrylamide gel electrophoresis and transferred to polyvinyl difluoride membranes (Trans-Blot Turbo System, Bio-Rad) for immunoblotting with the indicated antibodies as described previously.^27^ For the ubiquitination assay, the cell lysates were clarified by centrifugation at 12,000 × *g* for 5 min at 4 °C, followed by sequential incubation with an anti-MRTFA antibody overnight and protein A/G magnetic beads (Thermo Scientific, 88803) for 2 h at 4 °C. The beads were washed with Tris-buffered saline containing Tween-20 detergent (TBST) (Thermo Scientific, 28360) and then boiled in 2x loading buffer (Bio-Rad, 1610747) at 95 °C for 10 min. After magnetic separation, the supernatants were collected for Western blot analysis. Antibody information is provided in Supplementary Table 1.

### RNA isolation and qRT‒PCR

The peripheral adipose and adventitial layers were carefully removed from freshly isolated mouse aortas. Total RNA was extracted from the purified aortas using a miRNAeasy mini-Kit (QIAGEN, 217004). Three hundred nanograms (ng) of total RNA were used for cDNA synthesis via a reverse transcription kit (Bio-Rad, 1708891). qRT‒PCR was performed using iQ SYBR Green Supermix (Bio-Rad, 1708882) on a Real-Time PCR System (Bio-Rad, CFX384^TM^). The primer information is included in Supplementary Table 2.

### Transmission electron microscopy (TEM)

Freshly isolated AAs were fixed in 4% paraformaldehyde and 2% glutaraldehyde in 0.1 M sodium cacodylate (NaCac) buffer (pH 7.4), postfixed in 2% osmium tetroxide in NaCac, stained en bloc with 2% uranyl acetate, dehydrated with a graded ethanol series and embedded in Epon-Araldite resin. Thin sections (55 nm) were prepared using a diamond knife on an ultramicrotom (Leica EM UC7, Leica Microsystems) and collected on Synaptek copper slot grids. The grids were stained with 4% aqueous uranyl acetate and Reynolds lead citrate. The tissue sections were observed under a transmission electron microscope (JEM 1400Flash, JEOL) at 120 kV and imaged with a OneView CCD Digital Camera (Gatan) in the Augusta University histology core.

### Proximity ligation assay (PLA) and immuno-RNA fluorescence in situ hybridization assay

PLA was performed via a commercial kit according to the manufacturer’s instructions (Sigma‒Aldrich, DUO92101-1KT). Briefly, primary MASMCs were fixed with methanol for 15 min on ice. The cells were washed with PBS and incubated with blocking solution in a humidified chamber at 37 °C for 60 min. The cells were then incubated with primary antibodies overnight at 4 °C. Following PLA probe incubation, ligation, amplification, and final washes, the samples were mounted with mounting media containing DAPI (Sigma‒Aldrich, DUO82040). Images were acquired using a confocal microscope (LSM 900, Zeiss). Information on the primary and secondary antibodies is provided in Supplementary Table 1 Immuno-RNA fluorescence for the VSMC marker protein MYH11 and in situ hybridization of mRNA for *MYOCD* were performed on human AAA and non-AAA tissues via a commercial RNAscope kit and probe (Advanced Cell Diagnostics, ACD), as previously described.^72^ Human AAA and non-AAA aorta samples were provided by the Department of Vascular and Endovascular Surgery, Heart and Vascular Centre, Semmelweis University, under approval from the Regional, Institutional Scientific and Research Ethics Committee (111/2022) and the National Center for Public Health and Pharmacy (9882-8/2022/EÜIG) in Hungary. Patient information is included in Supplementary Table 3.

### Chromatin immunoprecipitation (ChIP) assay

The ChIP assay was performed following the Millipore chromatin immunoprecipitation protocol with minor modifications. Briefly, 2 × 10^7^ MOVAS cells were crosslinked with 1% formaldehyde, and the reaction was quenched with 125 mM glycine. The cells were washed twice with ice-cold PBS and lysed with 200 µL of lysis buffer (Millipore, Cat #20--163) containing a proteinase inhibitor cocktail (PIC) (Research Products International, Cat #P50600--1). The lysates were sonicated to generate DNA fragments between 200 and 1000 bp, and 20 µl of sheared DNA was reserved as input. The remaining lysate was centrifuged at 13,000 rpm for 10 min at 4 °C, diluted with ChIP dilution buffer containing protein inhibitor cocktail (PIC) (Millipore, Cat #20--153), and incubated overnight at 4 °C with an anti-SRF antibody (Cell Signaling Technology, 5147S) or a species-matched IgG control (Cell Signaling Technology, 2729S). After incubation with Dynabeads (Thermo Scientific, 11203D), the protein/chromatin complexes were precipitated, washed, and eluted with freshly prepared elution buffer. DNA was recovered after reverse cross-linking, column purification, and then PCR amplification of the predicted CArG box containing the *Bcl2* proximal promoter region. An intronic CArG box in *Cnn1*^73^ and the 3’ untranslated region (3’ UPR) of *Bcl2* lacking a predicted CArG box were used as positive and negative controls, respectively. The sequence information of the relevant primers is included in Supplementary Table 2.

### Generation of the *Bcl2* promoter and CArG mutant reporter and luciferase assays

Genomic DNA isolated from MOVAS cells was used to PCR amplify the proximal *Bcl2* promoter harboring a predicted consensus CArG box (CCTAAAAAGG). The amplified fragment (645 bp) flanking this CArG box was cloned and inserted into the pGL3 basic luciferase reporter. After confirming CArG box activity, site-directed mutagenesis was performed to substitute CCT with GTC using a commercial kit as previously described.^5,70^ A luciferase assay was conducted in PAC1 SMCs with a Dual-Luciferase Reporter Assay System (Promega, E1910) as previously reported.^8,56^ Briefly, PAC1 SMCs were cultured in 24-well plates at ~70% confluence and cotransfected with a luciferase reporter carrying the 645 bp proximal promoter of mouse *Bcl2* encompassing the predicted CArG element or its CArG mutant with either the SRF-VP16 or pcDNA3.1-MYOCD expression plasmid. Transfections were carried out via Lipofectamine 3000 (Thermo Scientific, L3000008). Twenty-four hours after transfection, luciferase activity was measured via a FLUOstar Omega plate reader (BMG LABTECH, 415--3383) according to the manufacturer’s instructions. The primer information is included in Supplementary Table 2.

### ChIP sequencing (ChIP-seq) and data analysis

We strictly followed a ChIP-seq protocol (v011014) to prepare chromatin DNA for ChIP-seq.^74^ Briefly, four plates (150 mm) of 90% confluent HCASMCs were harvested to obtain 2 × 10^7^ cells for each preparation. The cells were crosslinked with 1% formaldehyde and sonicated with a Bioruptor UCD-200 (Diagenode, Denville, NJ, USA) at a high magnitude for 15 min followed by a middle magnitude for 10 min to obtain chromatin fragments 100–500 bp in length. The chromatin complexes were incubated with magnetic beads coated with rabbit polyclonal SRF (G-20, sc-335; Santa Cruz Biotechnology) for 2 h prior to DNA sample retrieval. The ChIP DNA concentration and size were determined by a Qubit fluorometer (Thermo Fisher, Waltham, MA) and an Agilent Bioanalyzer 2100 (Agilent, Santa Clara, CA), respectively. The TruSeq ChIP-seq Sample Preparation Kit was used for next-generation sequencing library construction per the manufacturer’s protocols (Illumina, San Diego, CA). Briefly, up to 10 ng of ChIP DNA with an optimal size (200–600 bp) was subjected to end repair with subsequent 3‘ adenylation to create a 3’dA overhang suitable for adapter ligation. Illumina adapters were ligated to both ends of the DNA, purified by gel electrophoresis and amplified using 15 cycles of PCR with primers specific to the adapter sequences to generate amplicons of ~200–500 bp. Libraries were hybridized to the Illumina single-end flow cell and amplified via cBot (Illumina, San Diego, CA). Single-end reads of 100 nt were generated for each sample. The ChIP FASTQ files were aligned using the CLC Genomics Workbench software. Next, peaks were called on the aligned data with the same software. The resulting BED files were then exported and further analyzed in RStudio. Briefly, the GenomicRanges and GenomicFeatures packages were used to clean the data and generate a GRanges object. This object was subsequently assessed for motif sequences obtained from the JASPAR database. For SRF, a position frequency matrix (PWM) was generated using peak data from the SRF ChIP experiment and known binding rules for the SRF-CArG interaction.^75^ Finally, after retrieving regions corresponding to the motifs of interest, ChIPseeker was used to annotate these regions based on several factors, including their distance to the TSS, location within annotated gene models, and nearest gene symbol.

### Nuclei isolation, snRNA-seq and ATAC-seq library preparation, sequencing, data processing, and quality control

AAs were harvested from WT and *Mapk14* KO mice following Ang II infusion for seven days. Nuclei were isolated via the 10x Genomics Nuclei Isolation Kit according to the manufacturer’s protocol (10X Genomics, CG000505). High-quality nuclei suspensions (>10,000 nuclei per group) were obtained at a concentration of 4000 nuclei/μl from 3 pooled AAs per group. snRNA-seq and snATAC-seq libraries were prepared according to the user guide (10X Genomics, CG000338). Library quality and concentration were assessed via an Agilent 2100 Bioanalyzer and a Qubit Fluorometer and sequenced on a NextSeq 2000 (for snRNA-seq) and an Illumina NextSeq 550 (for snATAC-seq) platform with 150-bp paired-end reads.

### snRNA-seq and snATAC-seq data analysis

The raw snRNA-seq reads were processed via Cell Ranger (version 3.1.0) with default parameters and the mm10 2020-A-2.0.0 reference genome. Subsequent analyses were performed via the R package Seurat v5.^76^ Cells of low quality (<1000 or >5000 genes per cell or >10% mitochondrial genes in the cell) were excluded. The singleCellTK package was then used to identify and remove putative doublets via the “scds_hybrid_call” method.^35^ Gene expression data were normalized within each sample, followed by canonical correlation analysis (CCA) to integrate datasets across samples. Uniform manifold approximation and projection (UMAP) was used for dimensional reduction, and cell clusters were annotated based on expression of cell-type-specific markers.

Raw reads of snATAC-seq were processed using Cell Ranger ATAC (version 3.1.0) with default parameters for read filtering, alignment against the mm10 2020-A-2.0.0 reference genome, and barcode counting. The resulting outputs were then subjected to the R package Signac (1.13.0) for subsequent analysis.^38^ Cells with nCount_peaks <3000 or >15000, nucleosome_signal >4, or TSS enrichment <2 were removed. Putative doublets were identified and discarded via the scDblFinder R package (v 1.4.0) with default parameters.^77^ For dimensionality reduction, latent semantic indexing (LSI) was performed via the RunTFIDF and RunSVD functions in Signac. To correct for batch effects, we performed the integration using Seurat’s “FindIntegrationAnchors” and “IntegrateEmbeddings” functions. The integrated reduced dimensions were then used as inputs for clustering and UMAP analysis. A gene activity score matrix was generated via the GeneActivity function of Signac and incorporated into the Seurat object for cluster-based analysis and visualization. The “FindAllMarker” function was used to identify cluster-specific genes or accessible peaks. The chromVar package (https://greenleaflab.github.io/chromVAR/) was then utilized to compute TF activity via the “RunChromVAR” function in Signac. Seurat within the Signac framework was used to identify differentially active motifs across cell types and samples. The SRA number of the snRNA/ATAC-seq dataset is PRJNA1332774.

### Statistical analysis

All the data are presented as the means ± SEMs. For in vitro experiments, *n* = 3 denotes three independent biological replicates. For in vivo mouse studies, n indicates the number of individual mice per group. All the statistical analyses were performed with SPSS 29.0 software (IBM, NY, USA). Data normality was evaluated via the Shapiro‒Wilk test. For two-group comparisons, a two-tailed Student’s *t* test was applied to normally distributed data, and the Mann–Whitney *U* test was applied to nonnormally distributed data. For multiple group comparisons with one variable, one-way ANOVA followed by Tukey’s post hoc test (equal variances) or Dunnett’s T3 test (unequal variances) was used. For nonnormally distributed data, the Kruskal‒Wallis test was used, followed by Bonferroni correction. For multiple group comparisons with 2 variables, two-way ANOVA with Bonferroni correction was used. A probability value of *P* < 0.05 was considered statistically significant. The enrichment of SRF/CArG target genes was analyzed via ChIPseeker. The related *p* values were calculated via the cumulative hypergeometric test, and the –log (*p* value) is shown on the right *Y* axis of the figure to indicate statistical significance. Effect size was evaluated by the enrichment factor, defined as the ratio of observed to expected gene counts. A cutoff of 1.5 was used with the enrichment of at least 50% enrichment over random expectation.

## Supplementary information

Sigtrans_Supplementary_Materials

Original western blots

## Data Availability

All data supporting the findings of this study are available within this manuscript and its supplementary information. The snRNA/ATAC-seq and ChIP-seq datasets generated in this study have been deposited in the NCBI Sequence Read Archive (SRA; accession number PRJNA1332774) and the European Nucleotide Archive (ENA; accession number PRJEB102512), respectively.
