# Supplementary material for: MAPK14 converges on key transcriptional machinery to promote vascular smooth muscle cell degeneration in abdominal aortic aneurysm
Source: Signal Transduct Target Ther. 2026 Jan 12;11:17. doi: 10.1038/s41392-025-02540-0 (PMC12795861; doi:10.1038/s41392-025-02540-0)
Supplement: Supplementary file 1 — Sigtrans_Supplementary_Materials [file 41392_2025_2540_MOESM1_ESM.docx]

Supplementary Materials for

MAPK14 converges on key transcriptional machinery to promote vascular smooth muscle cell degeneration in abdominal aortic aneurysm

Xiaoliang Wu^1#*^, Chunhui Wang^1,2#†^, Nestor Ishimwe^1#^, Wei Zhang^1,3†^, Jaser Doja^1^, Shengshuai Shan^1^, Chunyu Ge^1^, Yong Sun^4^, Jinjing Zhao^5^, Micah Castillo^6^, Peter Sotonyi^7^, Gergo Gyurok^7^, Gabor Csanyi^8^, W. Bart Bryant^1^, Kunzhe Dong^9^, Yabing Chen^4^, Roberto Vazquez-Padron^10^, Joseph M. Miano^1^, Xiaochun Long^1*^

Correspondence to: Xiaoliang Wu ([xiwu@augusta.edu](mailto:xiwu@augusta.edu)) or Xiaochun Long ([Xlong@augusta.edu](mailto:Xlong@augusta.edu))

**This PDF file includes:**

Figures. S1 to S11

Tables S1 to S3


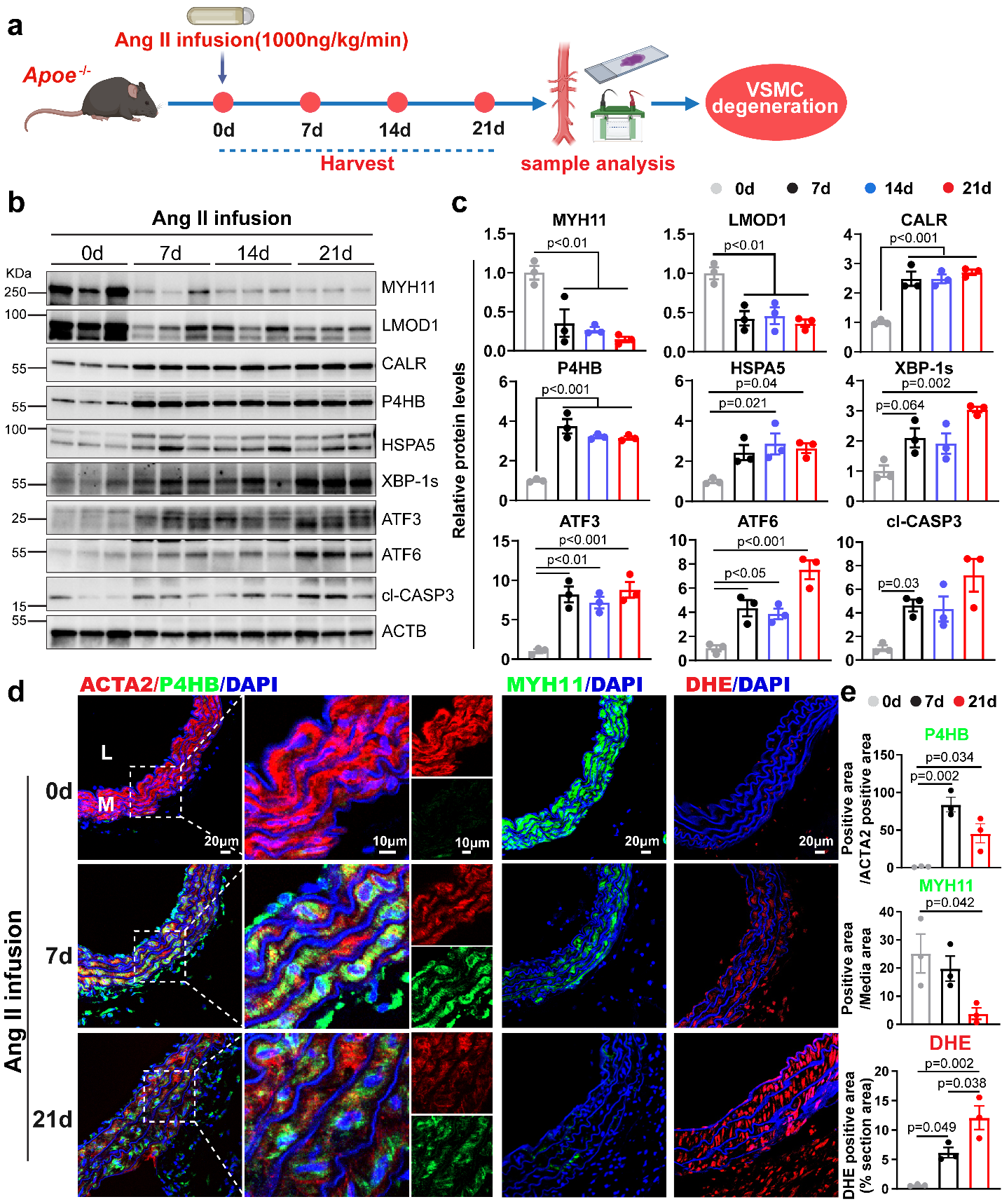
**Figure. S1. Activation of MAPK14 correlates with vascular smooth muscle cell (VSMC) degeneration during aortic aneurysm formation induced by Ang II.** **a** Schematic of the workflow for VSMC phenotype characterization in *Apoe*^-/-^ mice infused with Ang II for different times (Created with <https://www.biorender.com/>). **b** Representative Western blot images of the indicated proteins of the abdominal aortas (AAs) from the mice infused with Ang II for the indicated times. **c** Quantification of indicated proteins from panel **b** (n=3 per group). **d** Representative images of immunofluorescent staining of the indicated proteins and DHE staining in AAs after Ang II infusion. **e** The quantification of positive staining areas for the indicated proteins in the medial layer of AAs, and DHE-positive areas in the whole section area (n=3 per group). Data were analyzed using one-way ANOVA followed by either Tukey’s post hoc test for **c** (MYH11, LMOD1, CALR, P4HB, HSPA5, XBP-1s, ATF3, ATF6), **e** (MYH11, DHE), or by Dunnett T3’s post hoc test for **c** (cl-CASP3), and **e** (P4HB).


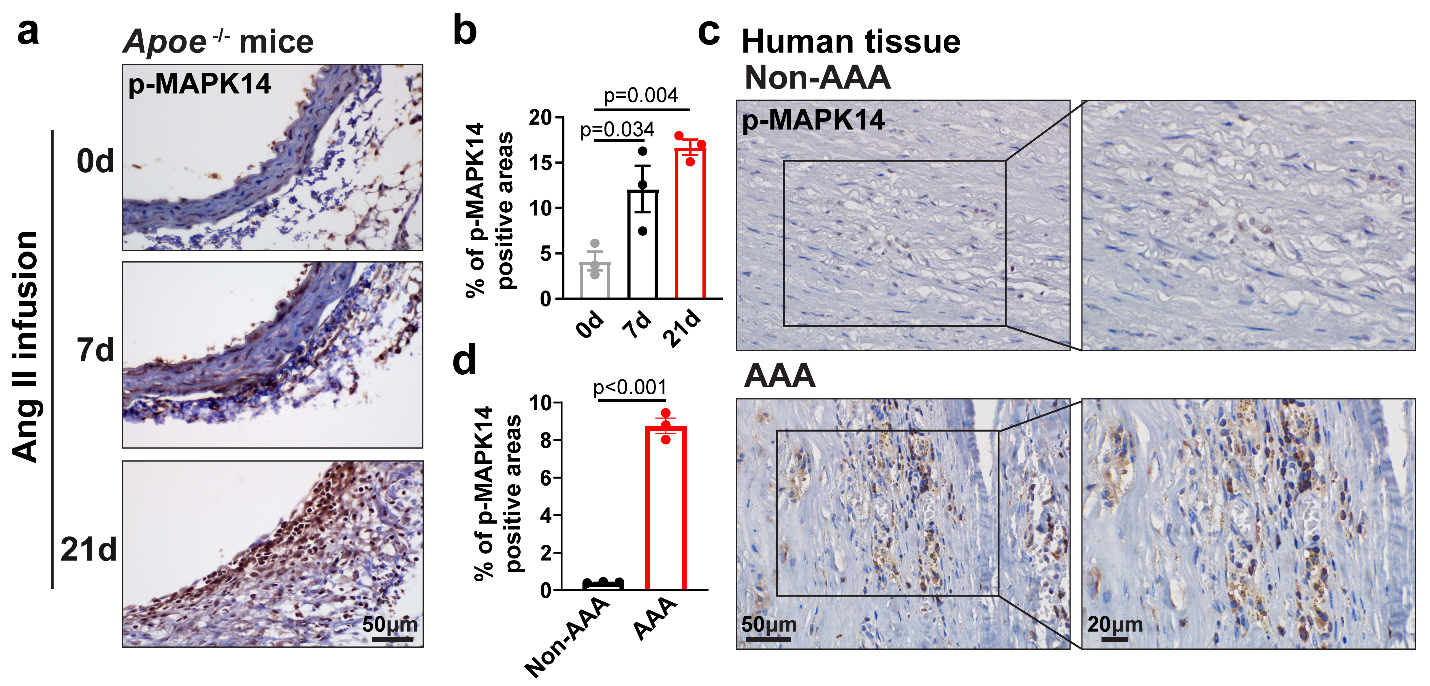
**Figure. S2. MAPK14 is activated in abdominal aortic aneurysm in Ang II mouse model and human tissues.** **a**, **b**, Representative DAB staining images of p-MAPK14 in abdominal aortas (AAs) after Ang II infusion for the indicated times (**a**) and the quantification of positive areas of p-MAPK14 (**b**) (n=3 per group). **c**, **d**, Representative DAB staining images of p-MAPK14 in human Non-AAA and AAA tissues (**c**) and the quantification of positive areas of p-MAPK14 (**d**) (n=3 per group). Data were analyzed using one-way ANOVA followed by Tukey’s post hoc test for **b** or using Student T-tests for **d**.


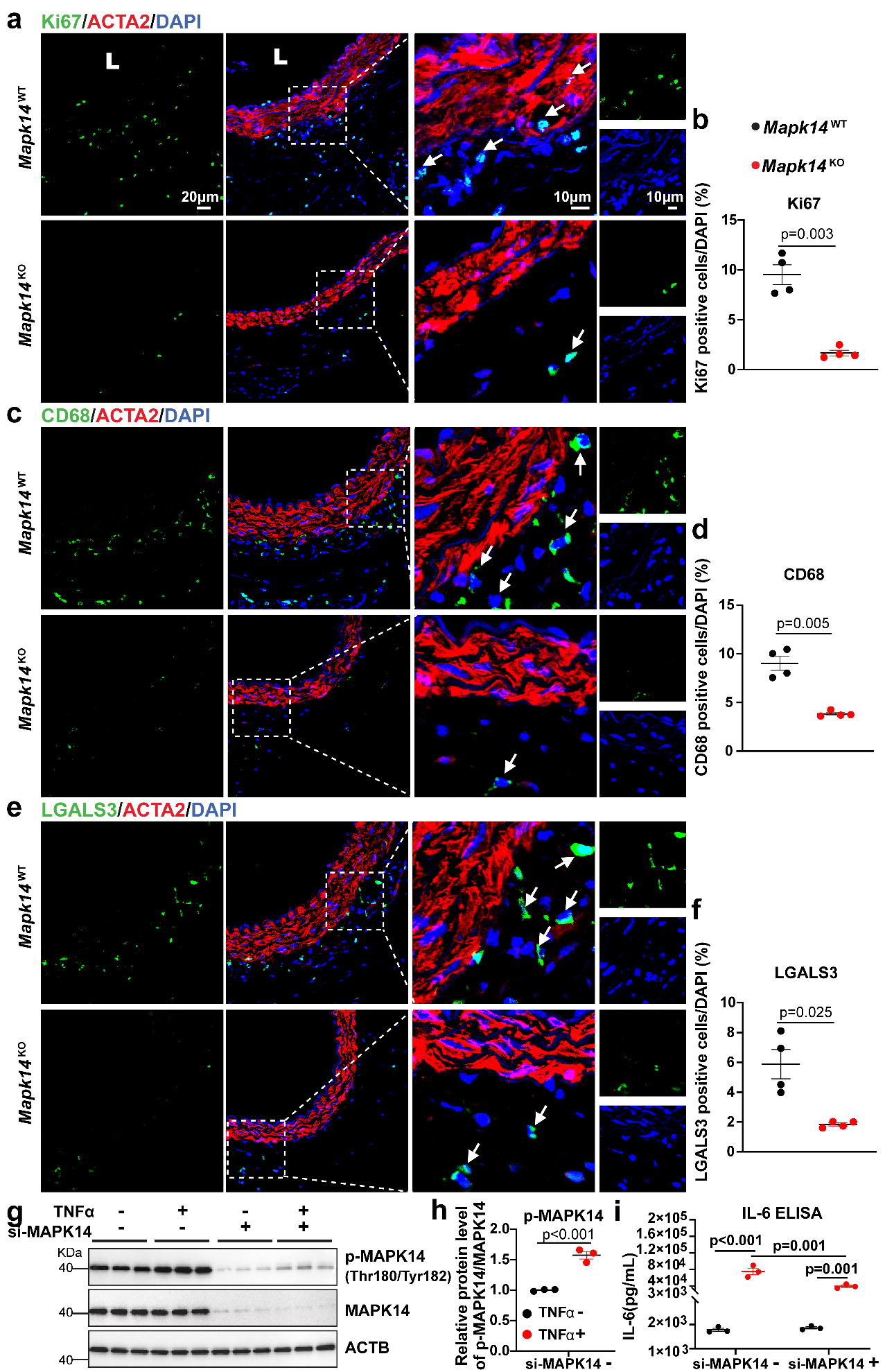


**Figure. S3.** **MAPK14 deficiency in VSMCs inhibits adventitial cell proliferation, reduces aortic macrophage infiltration, and decreases IL-6 production in HASMCs.** **a**, **b** Representative images of Ki67 staining in AAs of *Mapk*14^WT^ (*Mapk14*^f/f^/*Apoe*^-/-^) and *Mapk*14^KO^ (Sm22Cre^+/-^/*Mapk14*^f/f^/*Apoe*^-/-^) mice after Ang II infusion for 7 days (**a**) and the percentage of Ki67 positive cells relative to DAPI positive cells in the whole vessel wall (**b**) (n=4 per group). **c**, **d** Representative images of CD68 staining in AAs after Ang II infusion for 7 days (**c**) and the percentage of CD68 positive cells relative to DAPI positive cells in the whole vessel wall (**d**) (n=4 per group). **e**, **f** Representative images of LGALS3 staining in AAs after Ang II infusion (**e**) and the percentage of LGALS3 positive cells relative to DAPI positive cells in the whole vessel wall (**f**) (n=4 per group). **g** Representative Western blot images of MAPK14 under the indicated conditions. **h** Quantification of pMAPK14 from panel **g** (n=3). **i** ELISA quantification of IL-6 in culture supernatants under the indicated conditions (n=3). Data were analyzed using Student T-tests for (**b**, **d**, **f**, and **h**), or by two-way ANOVA followed by Bonferroni post hoc test for (**i**).


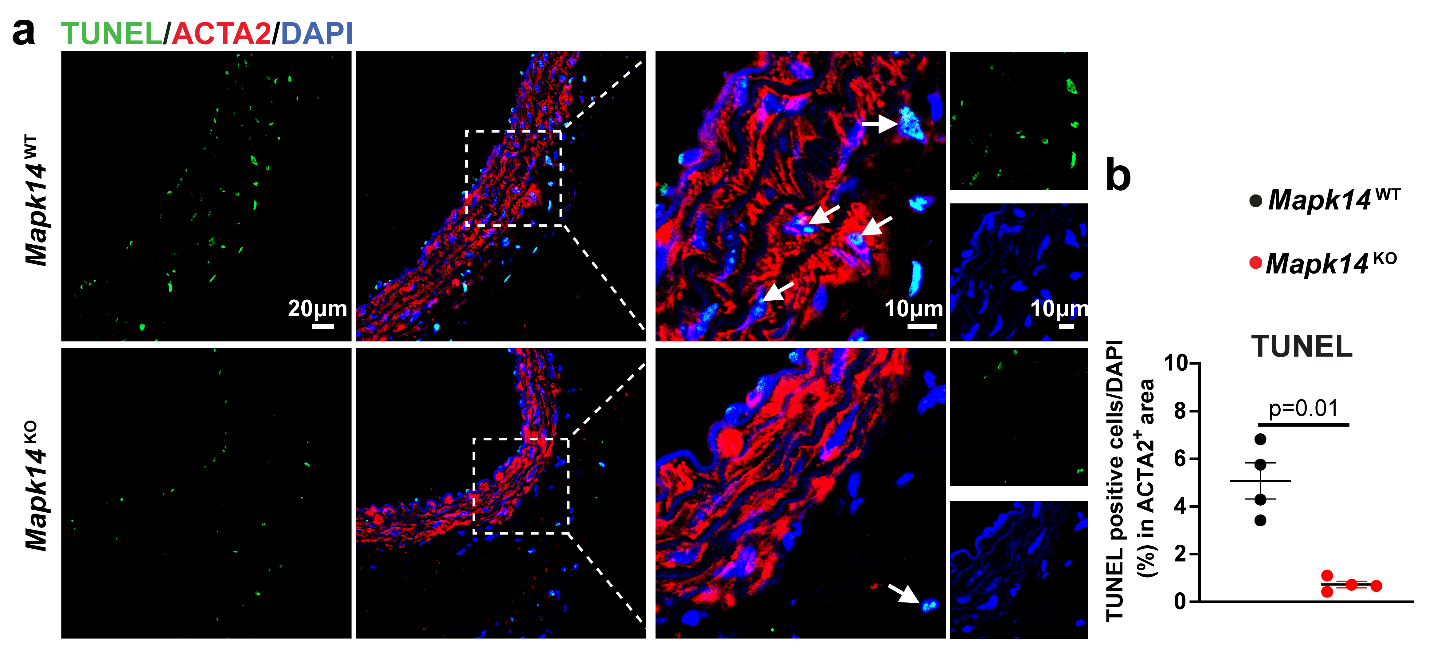
**Figure. S4. MAPK14 deficiency protects against Ang II-induced VSMC apoptosis.** **a**, **b** Representative images of TUNEL staining in AAs after Ang II infusion for 7 days (**a**) and the quantification of TUNEL-positive cells in ACTA2+ area (**b**) (n=4 per group). Data were analyzed using Student T-test.


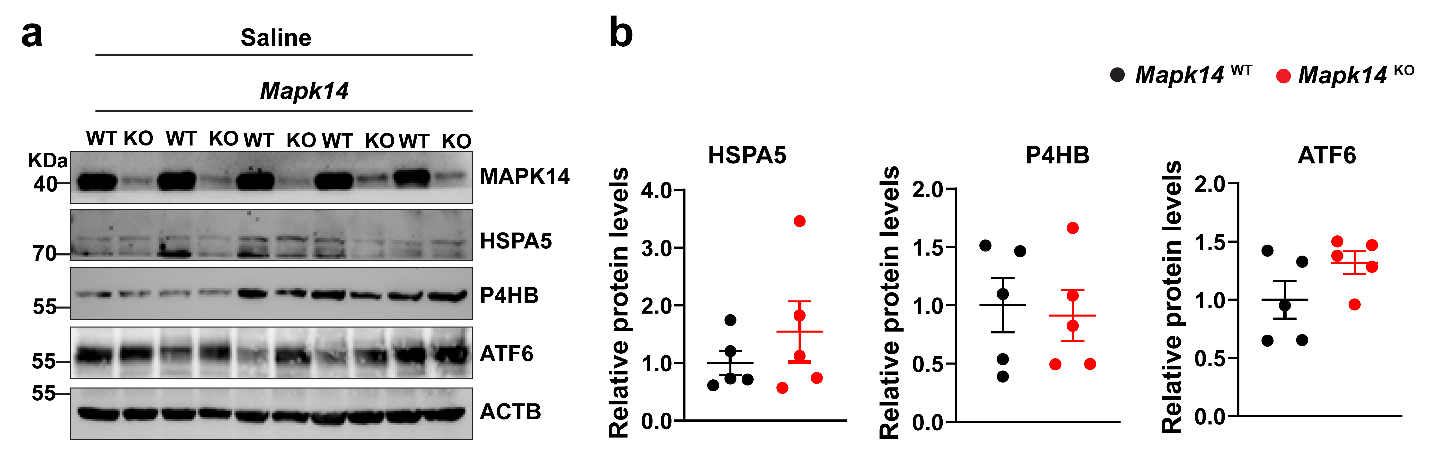
**Figure. S5. Loss of MAPK14 in VSMCs fails to alter the protein levels of ER chaperones in abdominal aortas from mice infused with saline. a** Representative Western blot images of the indicated proteins in *Mapk14* WT and *Mapk14* KO. **b** Quantification of indicated proteins from panel **a** (n=5 per group). Data was analyzed using Student T-test.


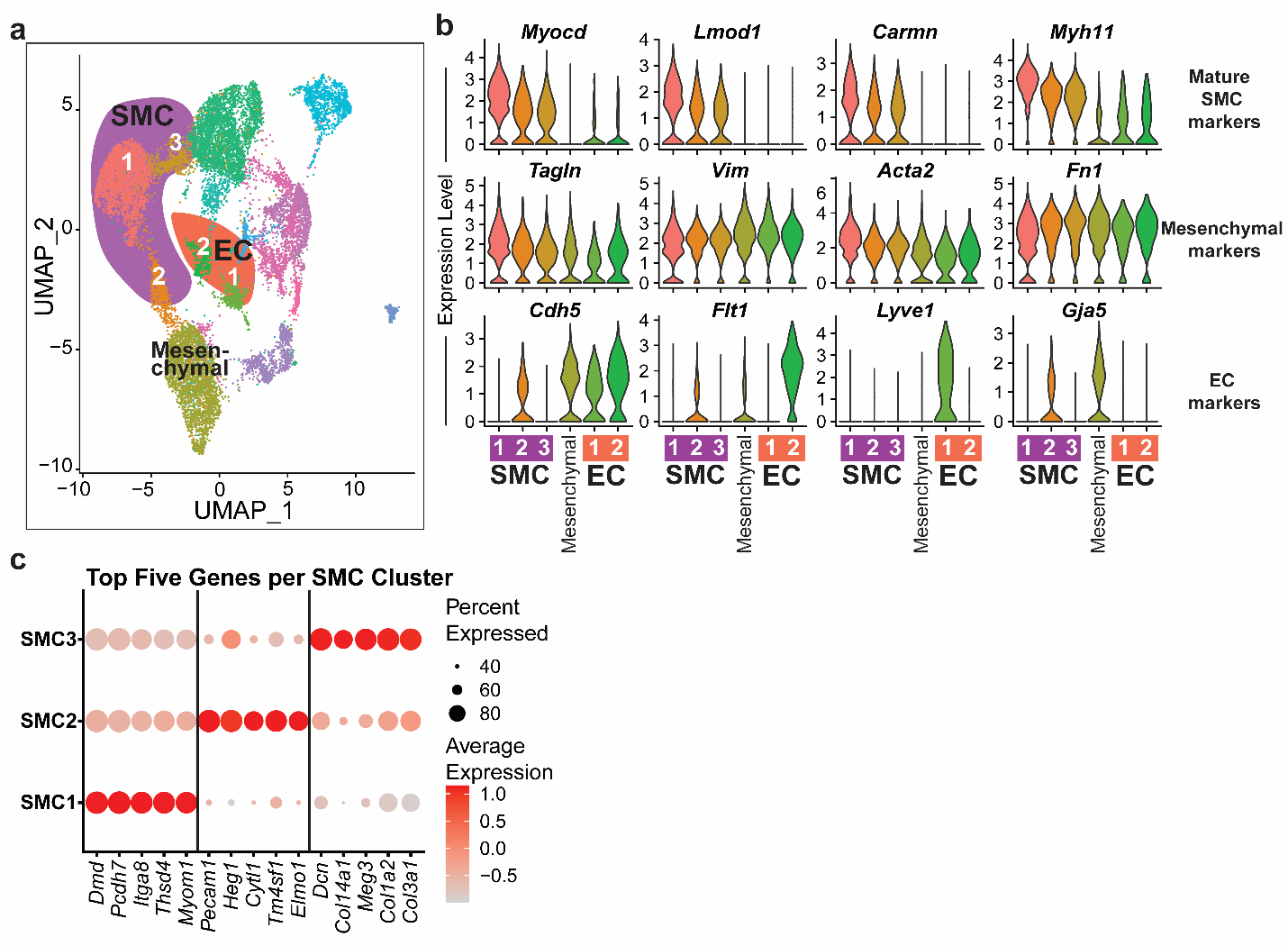
**Figure. S6. Marker gene expression profiles in smooth muscle cell (SMC), mesenchymal, and endothelial cell (EC) clusters. a** UMAP for SMC, Mesenchymal and EC populations. **b** Violin plots for gene expression of the indicated definitive SMC contractile, mesenchymal, and EC markers. **c** Top5 classification of the three VSMC clusters.


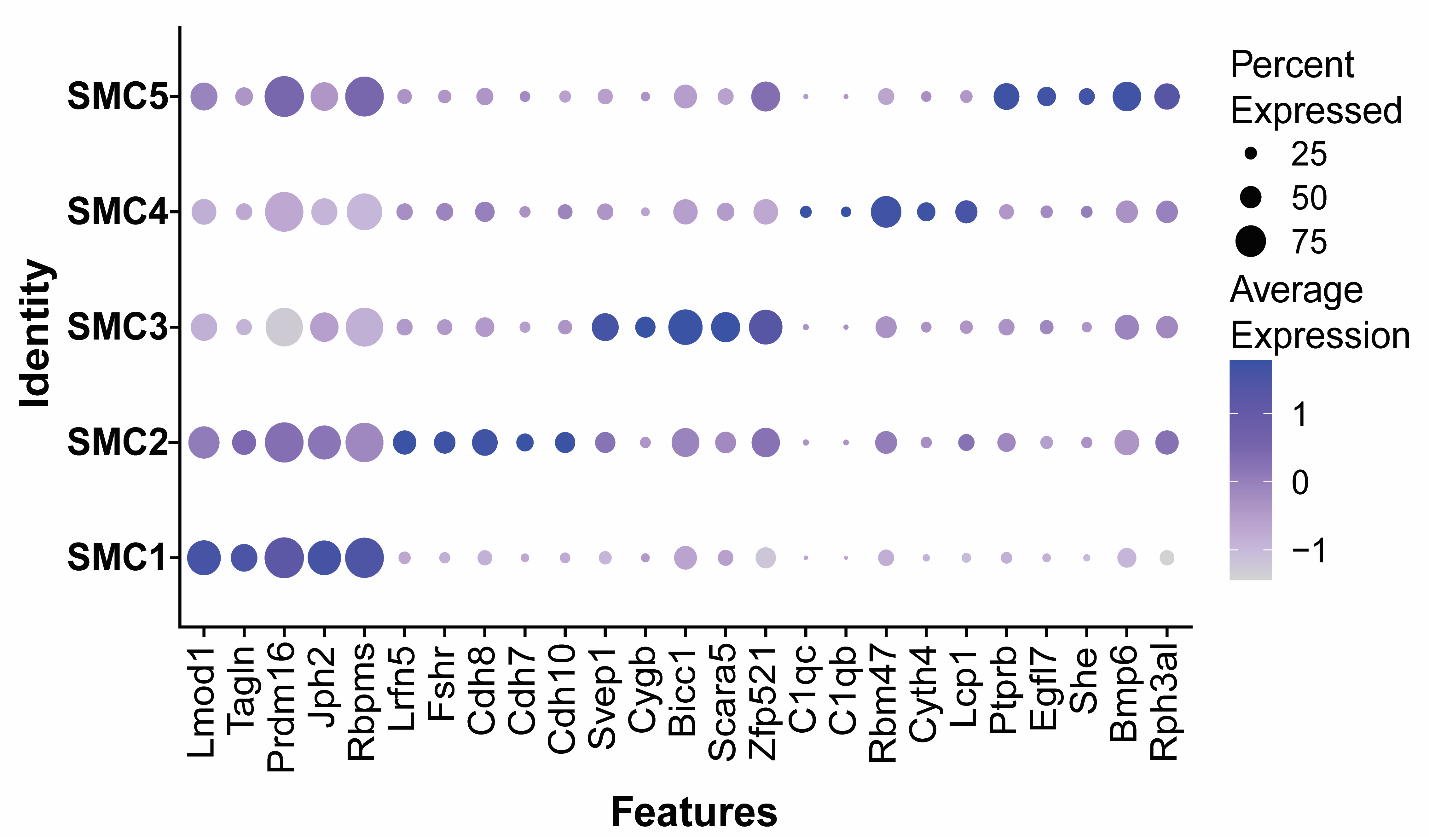
**Figure. S7. Top 5 to classify the 5 SMC clusters derived from snATAC-seq.**


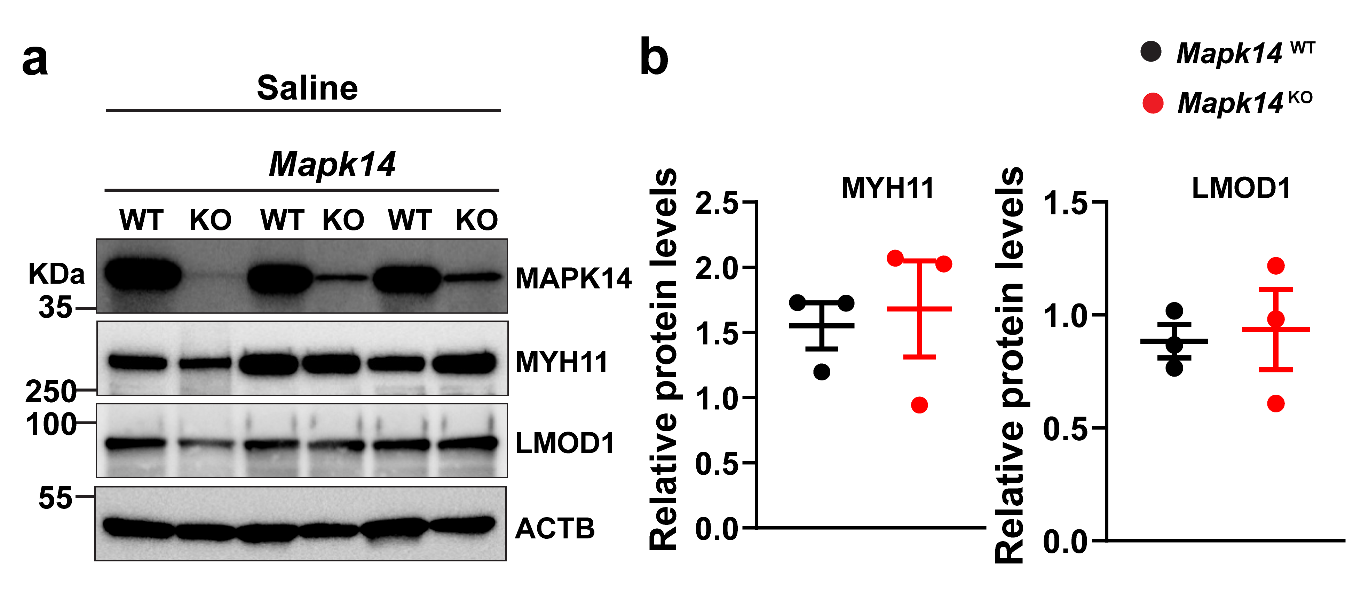
**Figure S8. Loss of MAPK14 in VSMCs fails to alter the protein levels of contractile markers in abdominal aortas from mice infused with saline. a** Representative Western blot images of indicated contractile proteins in AAs from *Mapk14* ^WT^ and *Mapk14* ^KO^ mice infused with saline for 7 days. **b** Quantification of indicated proteins from panel **a** (n=3 per group). Data were analyzed using Student T-tests.

**
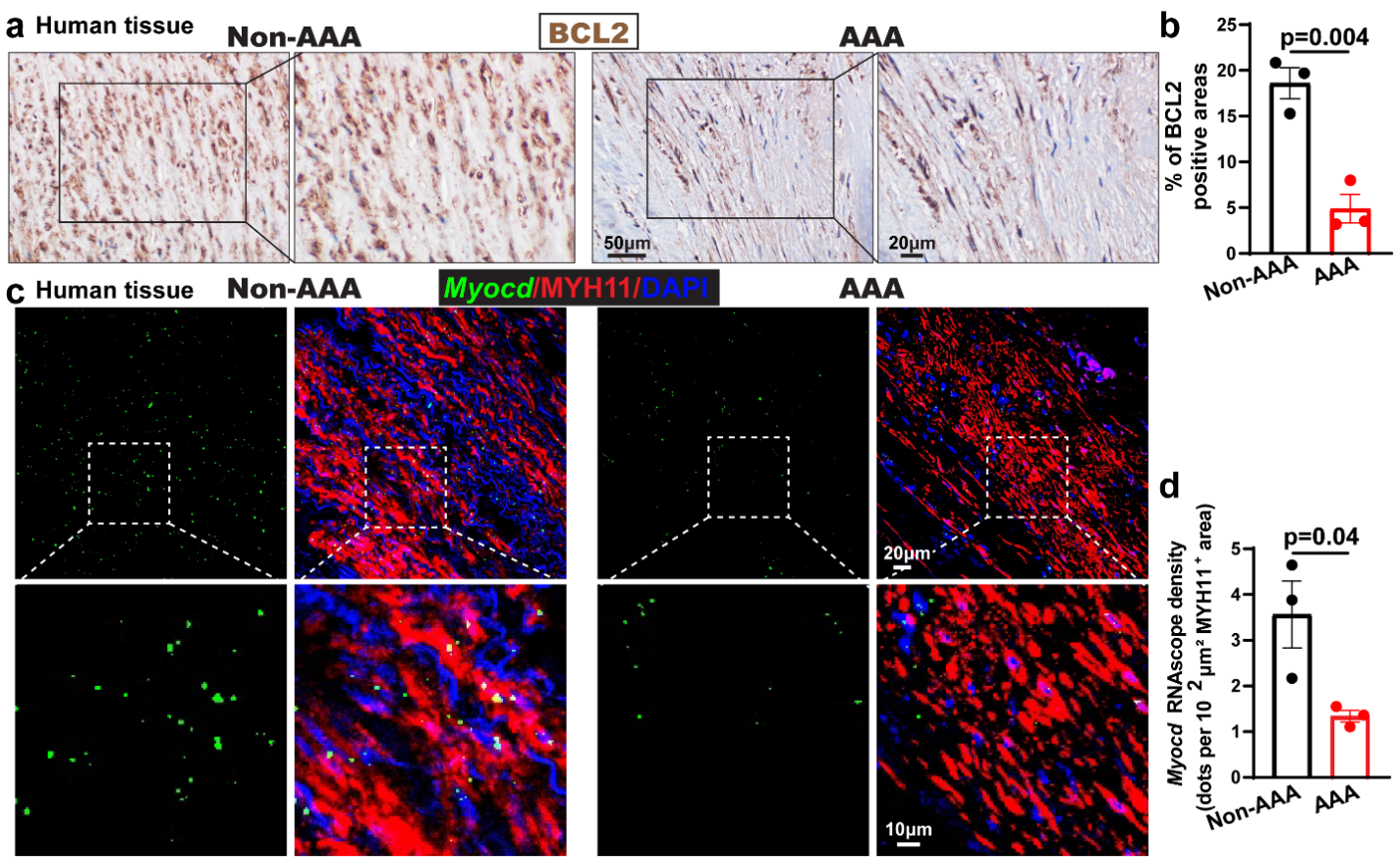
Figure. S9. Decreased expression of BCL2 protein and *MYOCD* mRNA in human AAA tissue**

**compared to non-AAA tissue**. **a** Representative DAB staining images of BCL2 in abdominal aortic aneurysm (AAA) tissue compared with non-aneurysmal aortic (Non-AAA) controls. **b** Quantification of BCL2 protein levels for panel **a** (n=3 per group). **c** Representative images of *MYOCD* RNAscope in situ hybridization in the above human tissues. **d** Quantification of *MYOCD* RNAscope density (RNAscope dots per 10^2^ μm^2^ MYH11^+^ area) (n=3 per group). Data were analyzed using Student T-tests.


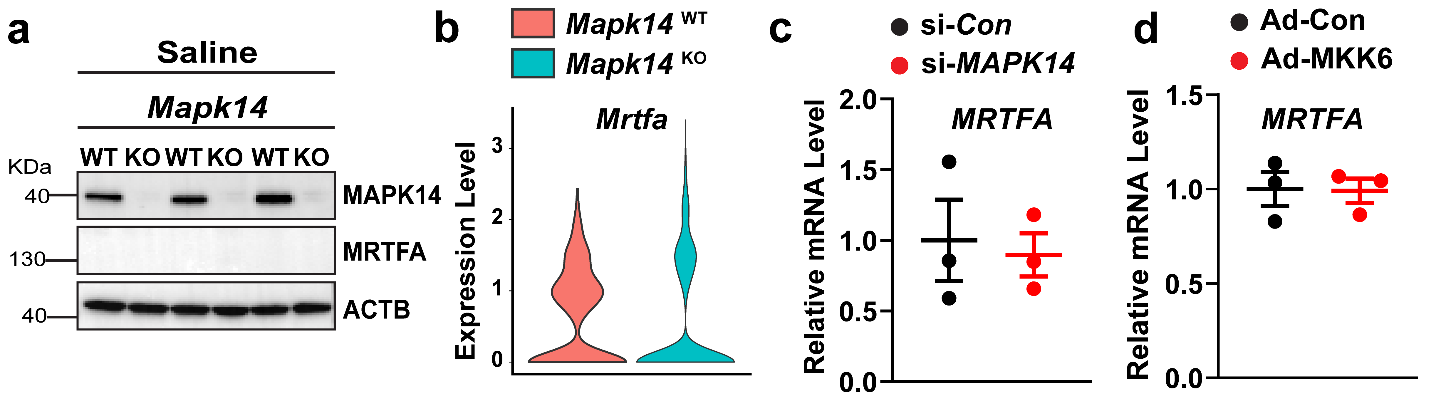
**Figure. S10. MAPK14 fails to influence *Mrtfa* mRNA in VSMCs. a** Western blot for MRTFA protein expression in saline-infused *Mapk14*^WT^ and *Mapk14*^KO^ mice (n=3 per group). **b** Violin plot showing *Mrtfa* gene expression in AAs from Ang II-infused *Mapk14* ^WT^ and *Mapk14* ^KO^ mice from snRNA-seq dataset. **c** qRT-PCR analysis of *MRTFA* gene expression in HASMCs transfected with si-*MAPK14* and si-*Con* (n=3). **d** qRT-PCR analysis of *MRTFA* gene expression in HASMCs ± Ad-MKK6 for 2 days (n=3). Data were analyzed using Student T-test.


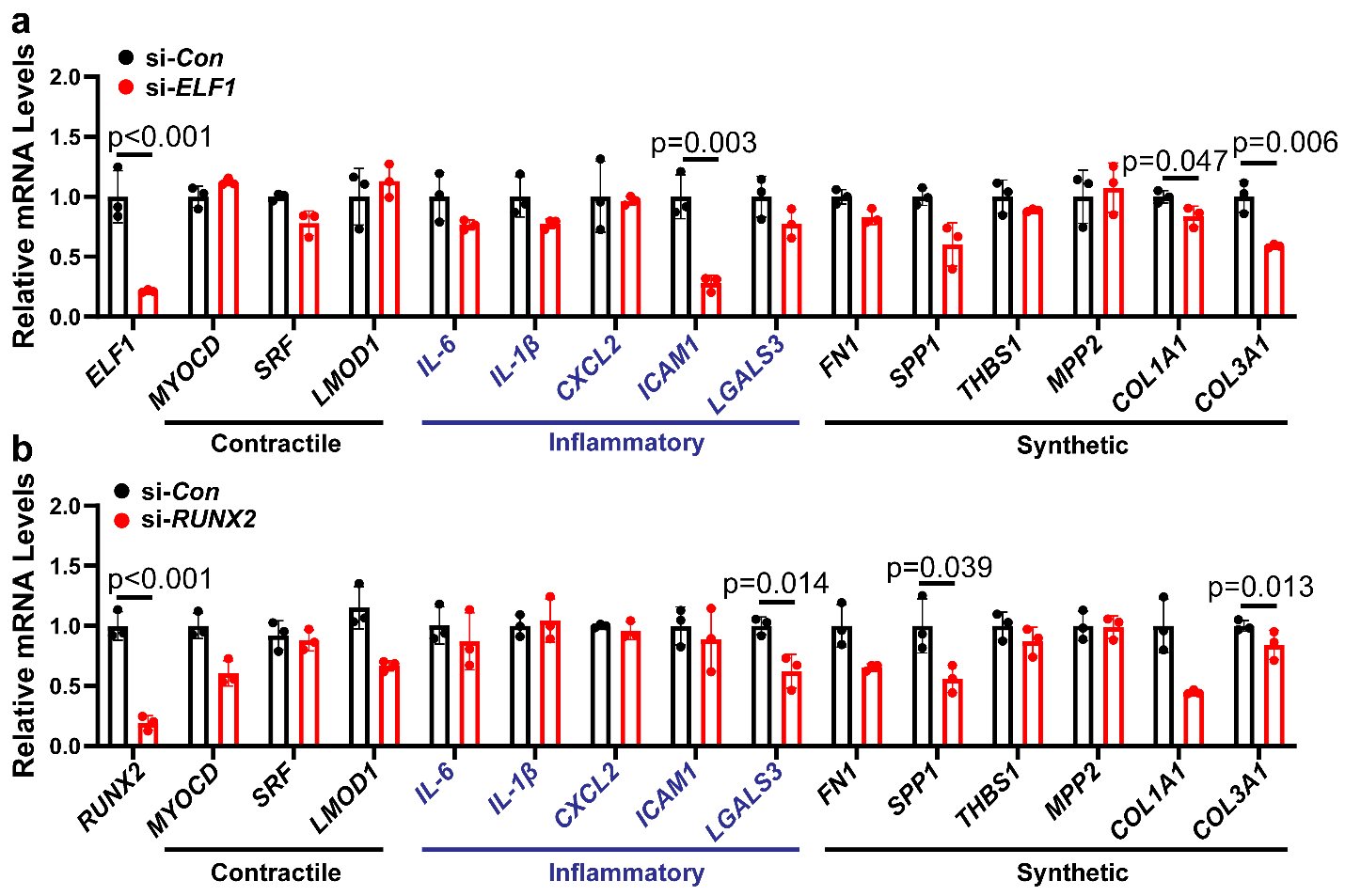
**Figure. S11.** **Effect of *ELF1* and *RUNX2* gene knockdown on VSMC degeneration in vitro. a, b** RT-qPCR analysis of the indicated VSMC contractile and degenerative markers (inflammatory and matrix remodeling genes) in HASMCs upon siELF1(**a**) and siRUNX2 (**b**) knockdown. Data were analyzed using Student T-tests.

**Supplementary Tables S1-S3**

**Table S1. Antibody information**

| **Antibody** | **Cat number** | **Work concentration** | **Supplier** |
| --- | --- | --- | --- |
|  |  |  |  |
| **Primary antibodies for Western blot** | | | |
| anti-MYH11 | ab224804 | 1:1000 | abcam |
| anti-LMOD1 | 15117-1-AP | 1:1000 | Proteintech |
| anti-CALR | ab92516 | 1:1000 | abcam |
| anti-P4HB | 3501S | 1:1000 | Cell Signaling |
| anti-HSPA5 | 3177S | 1:1000 | Cell Signaling |
| anti-XBP-1s | 12782S | 1:1000 | Cell Signaling |
| anti-ATF4 | 11815S | 1:1000 | Cell Signaling |
| anti-ATF6 | ab122897 | 1:1000 | abcam |
| anti-ATF3 | ab207434 | 1:1000 | abcam |
| anti-CASP3 | 9661S | 1:500 | Cell Signaling |
| anti-ACTB | A5441-100UL | 1:2000 | Sigma |
| anti-p38MAPK | A300-707A-T | 1:1000 | Bethyl Laboratories |
| anti-ACTA2 | A2547-100UL | 1:1000 | Sigma |
| anti-p-p38MAPK | 4511S | 1:1000 | Cell Signaling |
| anti-MRTFA | sc-390324 | 1:1000 | santa Cruz |
| anti-Ubiquitin | sc-8017 | 1:1000 | santa Cruz |
| anti-BCL2 | sc-7382 | 1:1000 | santa Cruz |
| anti-RUNX2 | D130-3 | 1:1000 | MBL Life Science |
| anti-MEF2D | 610774 | 1:1000 | BD Bioscience |
| anti-MEF2C | 5030S | 1:1000 | Cell Signaling |
| anti-SRF | 5147S | 1:1000 | Cell Signaling |
| anti-GAPDH | MAB374 | 1:2000 | EMD Millipore |
| Anti-USP10 | 8501S | 1:1000 | Cell Signaling |
| Anti-MYOCD | PA5-95700 | 1:1000 | Thermo Fisher Scientific |
| Anti-TUBA | T5168 | 1:2000 | Sigma |
| **Primary antibodies for immunostaining** | | | |
| anti-ACTA2 | C6198 | 1:500 | Sigma |
| Anti-Collagen I | ab270993 | 1:300 | abcam |
| anti-P4HB | 3501S | 1:200 | Cell Signaling |
| anti-p-p38MAPK | 4511S | 1:200 | Cell Signaling |
| anti-MYH11 | ab224804 | 1:200 | abcam |
| anti-HSPA5 | 3177S | 1:200 | Cell Signaling |
| anti-CASP3 | 9664S | 1:100 | Cell Signaling |
| Anti-CD68 | 97778S | 1:200 | Cell Signaling |
| Anti-Galectin 3 | 14-5301-82 | 1:200 | Thermo Fisher Scientific |
| Anti-Ki67 | 12075S | 1:400 | Cell Signaling |
| anti-BCL2 | ab182858 | 1:200 | Abcam |
| **Primary antibodies for immunoprecipitation** | | | |
| anti-MRTFA | A302-202A | 1:200 | Bethyl Laboratories |
| **Primary antibodies for PLA** | | | |
| anti-p-p38MAPK | 4511S | 1:200 | Cell Signaling |
| anti-MRTFA | sc-390324 | 1:150 | santa Cruz |
| **Secondary antibodies for Western blot** | | | |
| Goat anti-Mouse IgG (H+L) Secondary Antibody, HRP | 31430 | 1:10000 | Thermo Fisher Scientific |
| Goat anti-Rabbit IgG (H+L) Secondary Antibody, HRP | 31460 | 1:10000 | Thermo Fisher Scientific |
| VeriBlot for IP Detection Reagent (HRP) | ab131366 | 1:1000 | Abcam |
| **Secondary antibody for immunostaining** | | | |
| Goat anti Rabbit IgG (H+L) Secondary Antibody, Alexa Fluor 488, Invitrogen | A11008 | 1:200 | Thermo Fisher Scientific |
| Goat anti Rabbit IgG (H+L) Secondary Antibody, Alexa Fluor 647, Invitrogen | A21244 | 1:200 | Thermo Fisher Scientific |

**Table S2. Primer sequence information**

| **Primers used in qRT-PCR** | |
| --- | --- |
| Gene | Primers (5'-3') |
| *Srf* (Mus musculus) | F: CCACCACAGACCAGAGAATGAG |
|  | R: TCTTGAGCACAGTCCCGTTG |
| *Runx1*(Mus musculus) | F: TACTCGGCAGAACTGAGAAATG |
|  | R: GTAAAGACGGTGATGGTCAGAG |
| *Runx2(Mus musculus)* | F: CCAACCGAGTCATTTAAGGCT |
|  | R: GCTCACGTCGCTCATCTTG |
| *Myocd* (Mus musculus) | F: AAGGTCCATTCCAACTGCTC |
|  | R: CCATCTCTACTGCTGTCATCC |
| *Myh11*(Mus musculus) | F: TGCCGACACAGCCTACAGAAG |
|  | R: GGACGCCACCACAGCCAAG |
| *Itga8* (Mus musculus) | F: TGACACCACCAACAACAGG |
|  | R: AGTTCTCCAGTGATACAAAGGG |
| *Tagln* (Mus musculus) | F: AAGCCTTCTCTGCCTCAAC |
|  | R: ACCATTCTTCAGCCACACC |
| *Bcl2* (Mus musculus) | F: TTTGAGTTCGGTGGGGTCAT |
|  | R: CTGGGGCCATATAGTTCCACAA |
| *Mrtfa* (Mus musculus) | F: ACGAGGCGGTTACCATCAC |
|  | R: GCAGACAGAGACAGGAGCAC |
| *Col1a1* (Mus musculus) | F: TTCGTGACCGTGACCTTGAG |
|  | R: CCAGGTTGCAGCCTTGGTTA |
| *Col3α1* (Mus musculus) | F: GCCACCTTGGTCAGTCCTAT |
|  | R: GAAGCACAGGAGCAGGTGTA |
| *Cthrc1 (*Mus musculus) | F: CCCATCGAAGCCATCATCTATC |
|  | R: TCTACCAATCCAGCACCAATC |
| *Pi16* (Mus musculus) | F: GCACGAGGAGCATGAGTATTA |
|  | R: CTCAGTCTTGCTCCACACTAC |
| *Thbs1* (Mus musculus) | F: CCACAGTTCCTGATGGTGAAT |
|  | R: CCACGTTGCTGAATTCCATTG |
| *Hsp90ab1*(Mus musculus) | F: TTCTATAAGAGCCTCACCAATGAC |
|  | R: CCTGAATTCCAACTGACCTTCT |
| *Calr* (Mus musculus) | F: GACATGCATGGAGACTCAGAATA |
|  | R: AGCACATTCTTGCCCTTGTA |
| *18s* (Mus musculus) | F: ATGCGGCGGCGTTATTCC |
|  | R: GCTATCAATCTGTCAATCCTGTCC |
| *BCL2* (Homo sapiens) | F: GTGGATGACTGAGTACCTGAAC |
|  | R: GCCAGGAGAAATCAAACAGAGG |
| *MRTFA* (Homo sapiens) | F: CAGCCTGAAGGAAGCCATC |
|  | R: GCCCATCGGAAGTTGAGAC |
| *ELF1* (Homo sapiens) | CCAGTACCATGCAGGATGAA |
|  | GAGACACAACCACTGGAACT |
| *RUNX2* (Homo sapiens) | AGCAAGGTTCAACGATCTGAG |
|  | CCGAGGTCCATCTACTGTAAC |
| *MYOCD* (Homo sapiens) | TGCTGCTGTAAAGTCCAAATCC |
|  | GCGTAGGCTGAGTCCATAGG |
| *SRF* (Homo sapiens) | ACAGACCTCACGCAGACCTC |
|  | CAGTTGTGGGCACGGATGAC |
| *LMOD1* (Homo sapiens) | GCGGCAGAGAAACCAGAC |
|  | CCACTTGCTTGCTTTCATCC |
| *IL6* (Homo sapiens) | GTGTTGCCTGCTGCCTTC |
|  | AGTGCCTCTTTGCTGCTTTC |
| *IL1B* (Homo sapiens) | AGGCACAAGGCACAACAG |
|  | GTGGTCGGAGATTCGTAGC |
| *CXCL2* (Homo sapiens) | GCATCGCCCATGGTTAAGA |
|  | TCAGGAACAGCCACCAATAAG |
| *ICAM1* (Homo sapiens) | GATGGGCAGTCAACAGCTAAA |
|  | GGTAAGGTTCTTGCCCACTG |
| *LGALS3* (Homo sapiens) | GGGAAGAAAGACAGTCGGTTT |
|  | AACCTTGAAGTGGTCAGGTTC |
| *FN1* (Homo sapiens) | GGCACTGATGAAGAACCCTTAC |
|  | CTTATGCCTCTGCTGGTCTTTC |
| *SPP1* (Homo sapiens) | CATATGATGGCCGAGGTGATAG |
|  | AGGTGATGTCCTCGTCTGTA |
| *THBS1* (Homo sapiens) | GGTTGGAGATCAGTGTGACAA |
|  | GTATCTCCAATGCGGTCTGAG |
| *MMP2* (Homo sapiens) | CACAGCCAACTACGATGATG |
|  | AAGGTCAATGTCAGGAGAGG |
| *COL1A1* (Homo sapiens) | GTCACCCACCGACCAAGAAACC |
|  | AAGTCCAGGCTGTCCAGGGATG |
| *COL3A1* (Homo sapiens) | ATGCCCTACTGGTCCTCAGA |
|  | GGAACCAGGATGACCAGATG |
| *18S* (Homo sapiens) | F: ATGGGCGGCGGAAAATAGC |
|  | R: TCTTGGTGAGGTCAATGTCTGC |
| **Primers used in ChIP-PCR** | |
| *Bcl2* promoter with CArG box (Mus musculus) | F: CCGCAGCGGAGGAGGAGAAAG |
|  | R: AAGGAGCAGCCCGCGCAC |
| *Bcl2* 3-UTR without CArG box (Mus musculus) | F: GCAAAAGGTTCACTAAAGCAGT |
|  | R: CGGTTTTCAAAGCCTGGAGT |
| *Cnn1* intron 1 with CArG box (Mus musculus) | F: AAGGGCCGGTTTGCTTTA |
|  | R: TGG GGA GAG GAG GGA TGC AGC |
| **Primers used for luciferase reporter construction and mutagenesis** | |
| *Bcl2* WT promoter (Mus musculus) | F: GATACGGTACCCTCACACGCCCACTGAGCCA |
|  | R: GATACCTCGAGAAGGAGCAGCCCGCGCAC |
| *Bcl2* CArG mutagenesis primers (Mus musculus) | F: ACTTCGTAGCAGTCATgtcTTTTAGGAAAAAGAGGGGGGG |
|  | R: CCCCCCTCTTTTTCCTAAAAgacATGACTGCTACGAAGT |

**Table S3. Human AAA and control patient information**

| **Sample ID** | **Condition** | **Age** | **Sex** | **BMI** | **Hypertension** | **Smoking** | **Medical Notes** |
| --- | --- | --- | --- | --- | --- | --- | --- |
| 2024-pa10-3101 | Control | 50 | Male | 29 | Yes | Yes | NA |
| 2024-pa10-10056 | Control | 60 | Male | 32 | No | No | NA |
| 12080_24 | Control | unknown | Male | NA | NA | NA | NA |
| 2022-pa10-19366 | AAA | 66 | Male | NA | Yes | NA | Myocarditis (scarlet fever complication); hypertension; hyperlipidemia; COPD; chronic bronchitis; chronic alcohol use. Surgeries: tonsillectomy, pelvic fistula, hysterectomy |
| 2022-pa10-8789 | AAA | 67 | Female | NA | Yes | NA | Hypertension; diabetes mellitus; hyperlipidemia; hypothyroidism; benign prostatic hyperplasia; status post umbilical hernioplasty |
| 2023-pa10-11720 | AAA | 72 | Male | NA | Yes | NA | NA |
