# Supplementary figures and images for: MAPK14 converges on key transcriptional machinery to promote vascular smooth muscle cell degeneration in abdominal aortic aneurysm
Source: Signal Transduct Target Ther. 2026 Jan 12;11:17. doi: 10.1038/s41392-025-02540-0 (PMC12795861; doi:10.1038/s41392-025-02540-0)

**Fig. 1g**

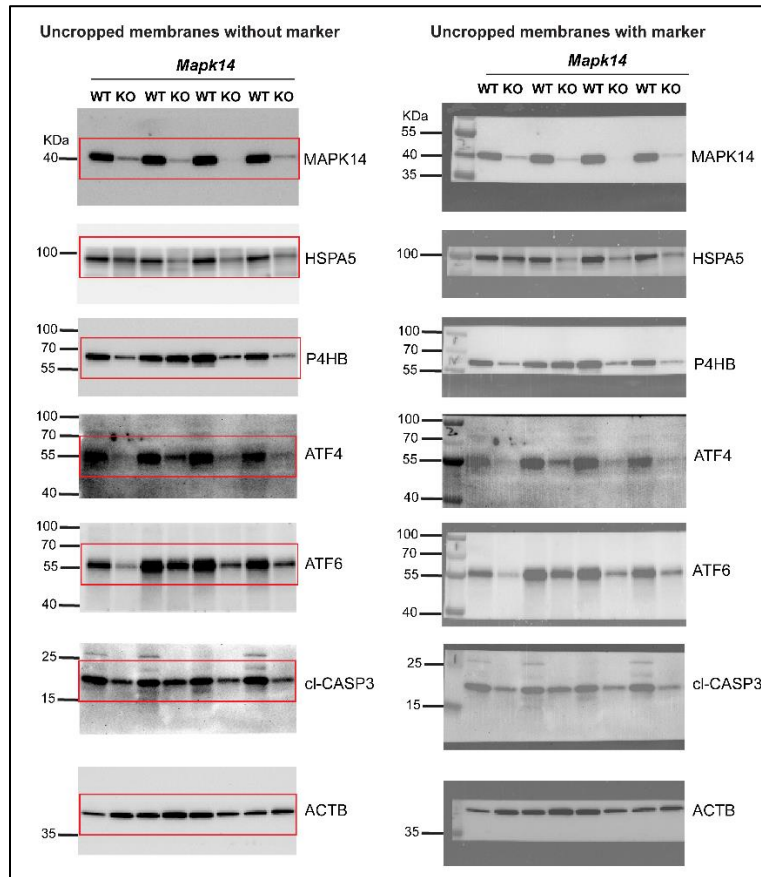

**Fig. 4e**

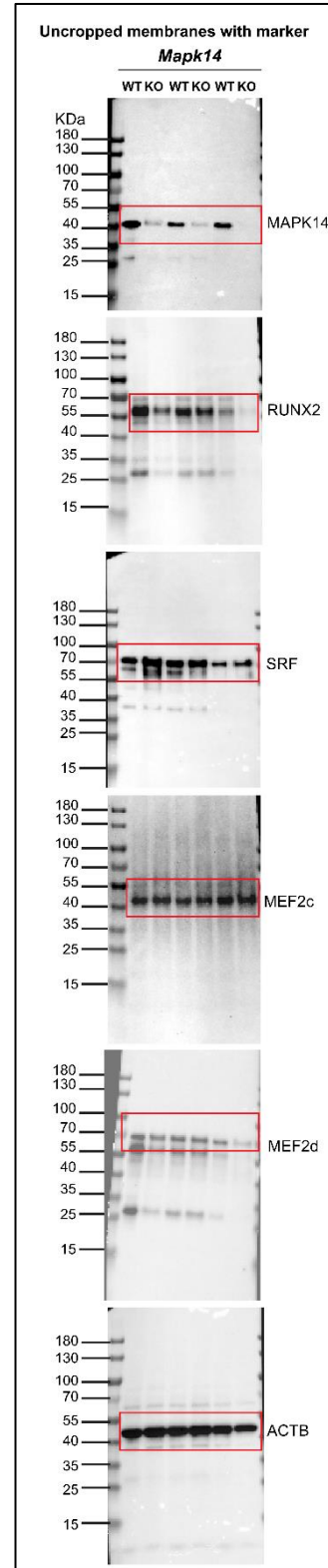

**Fig. 5j**

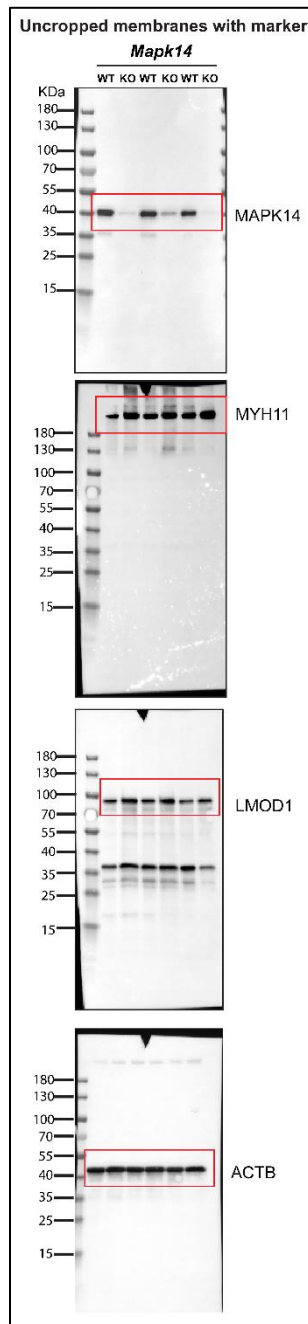

**Fig. 6e**

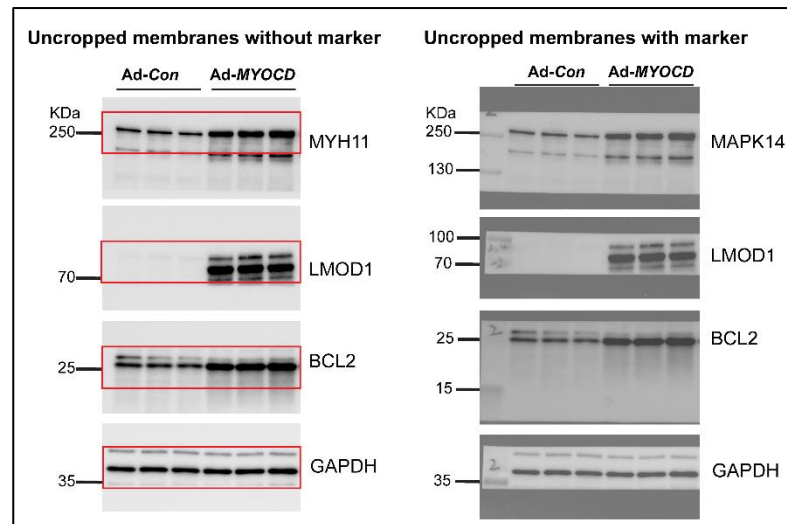

**Fig. 6f**

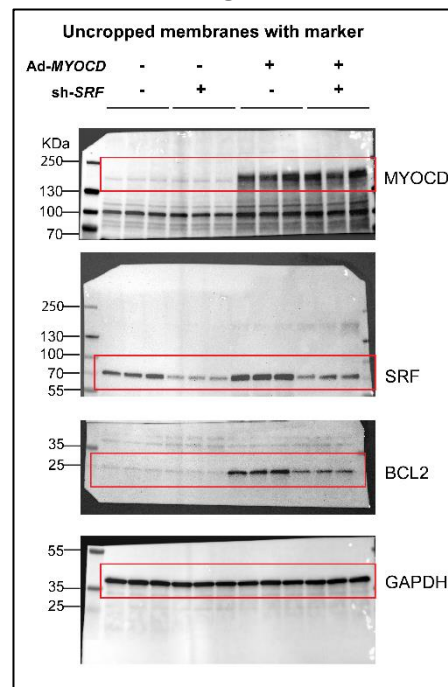

**Fig. 7d**

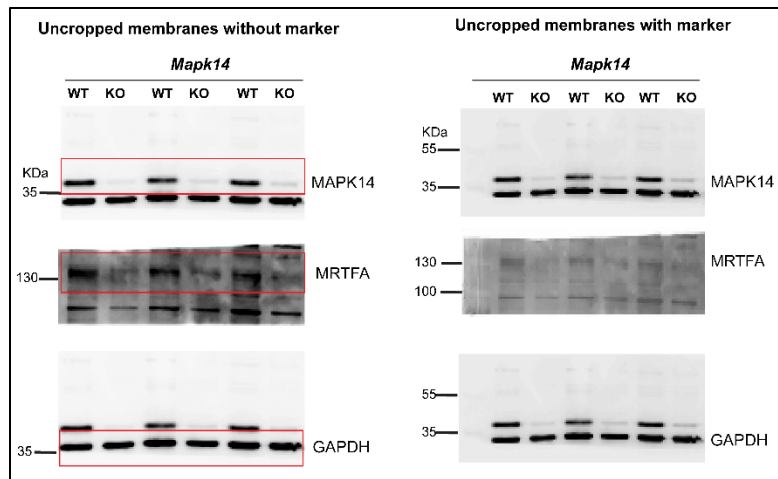

**Fig. 7e**

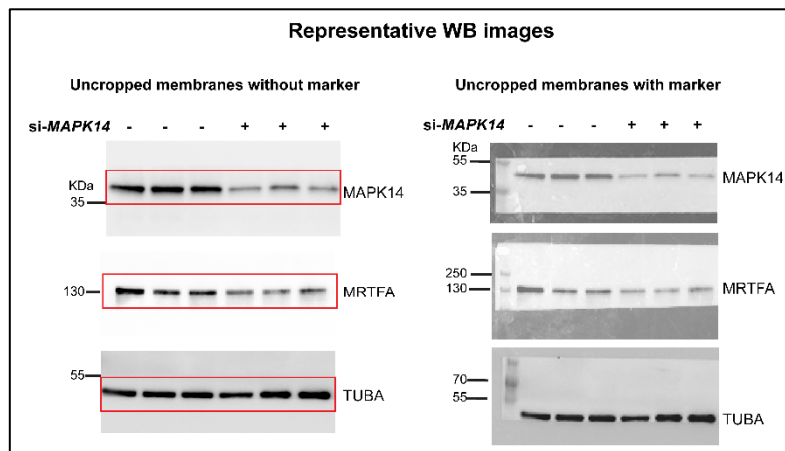

**Fig. 7f**

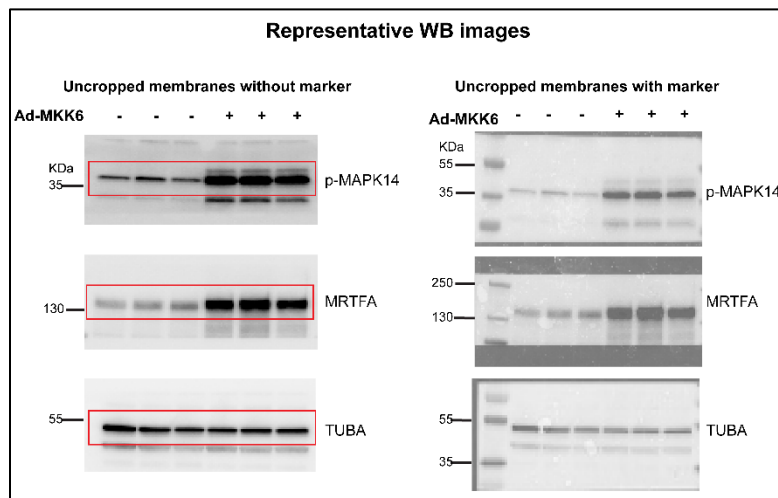

**Fig. 7g**

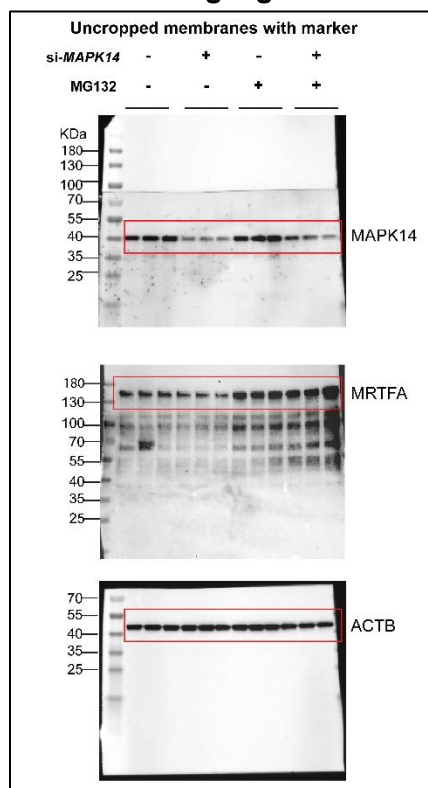

**Fig. 7h**

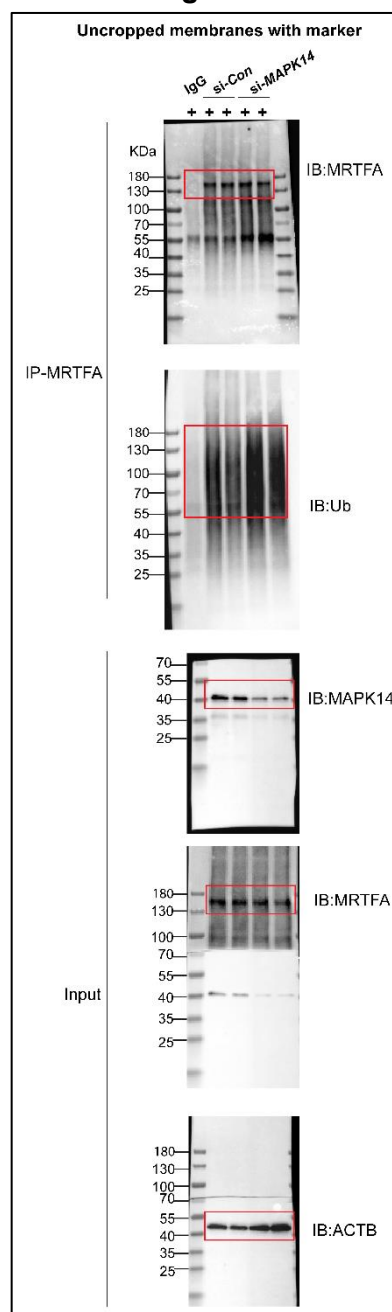

**Fig. 7k**

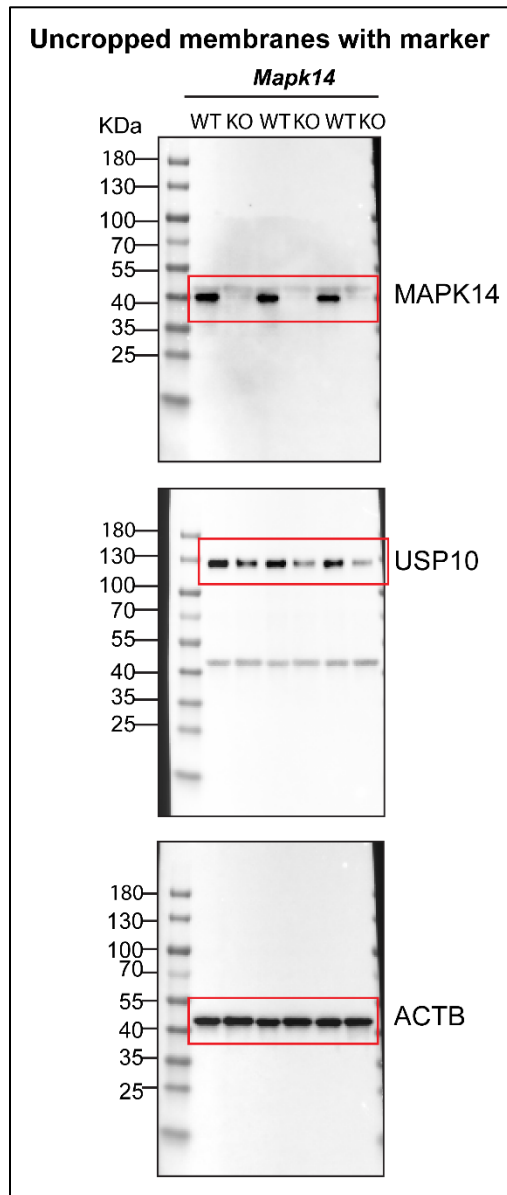

**Fig. 7l**

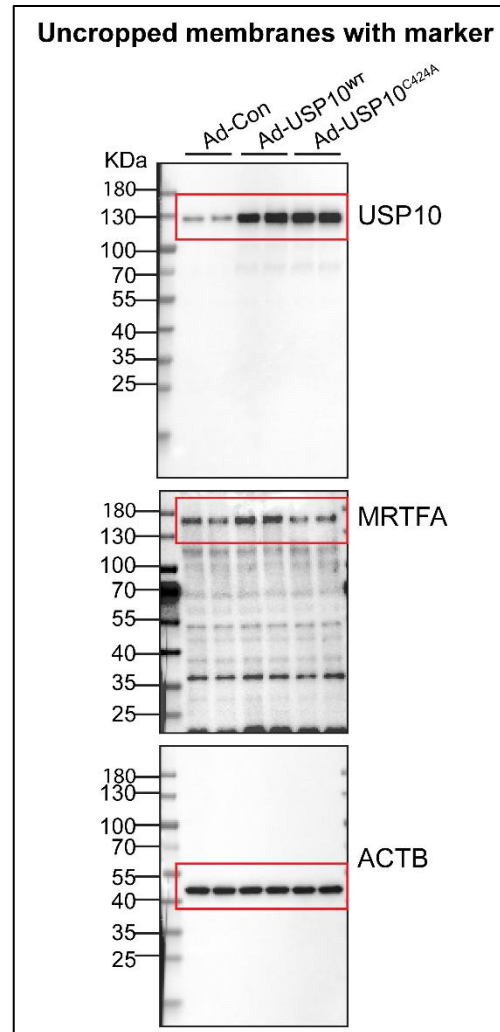

**Fig. 7m**

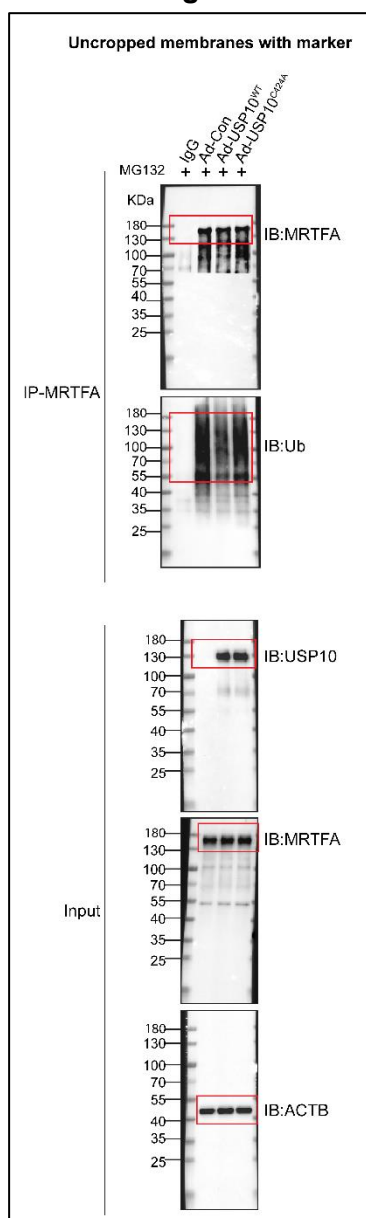

**Fig. S1b**

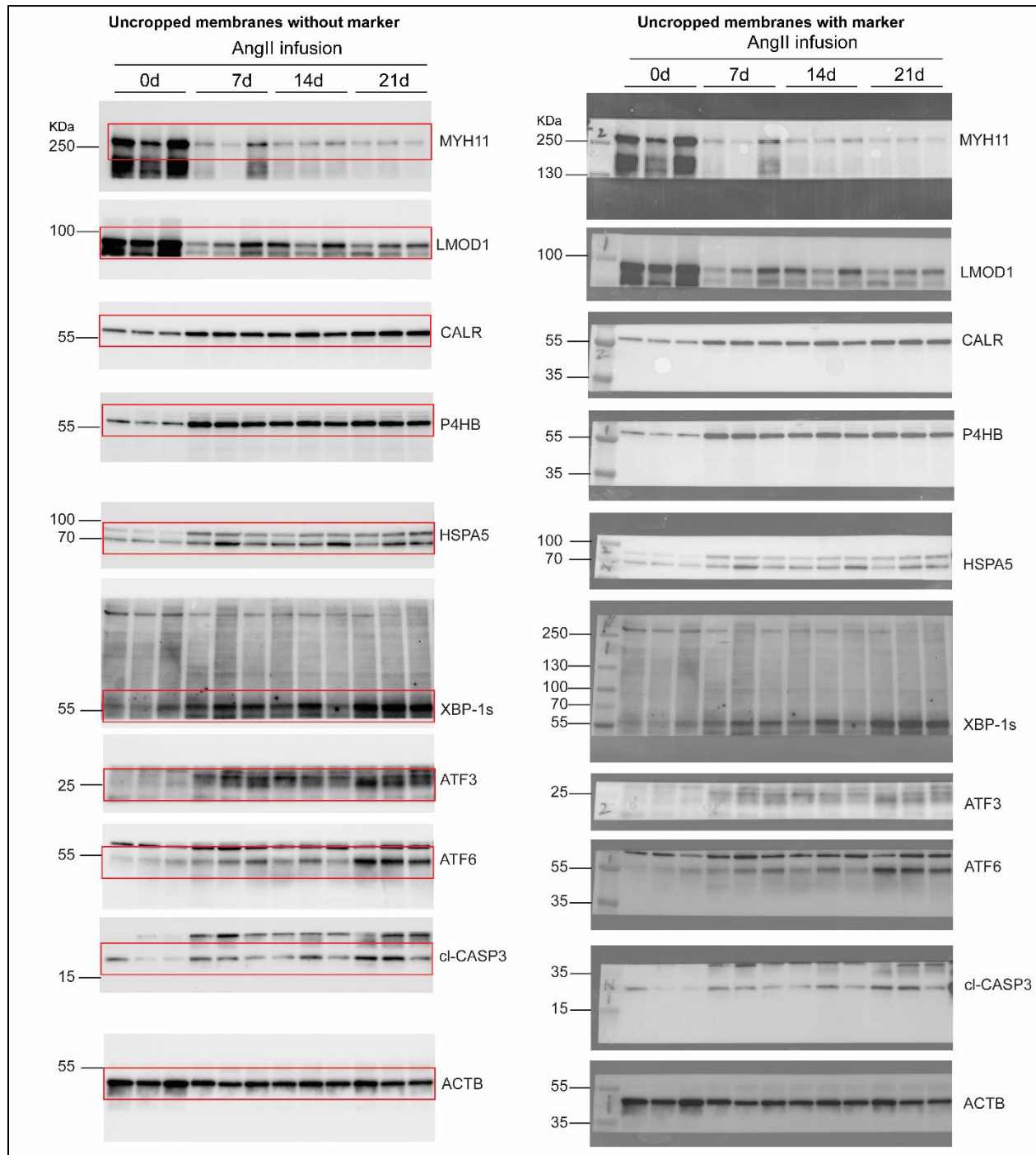

Fig. S3g

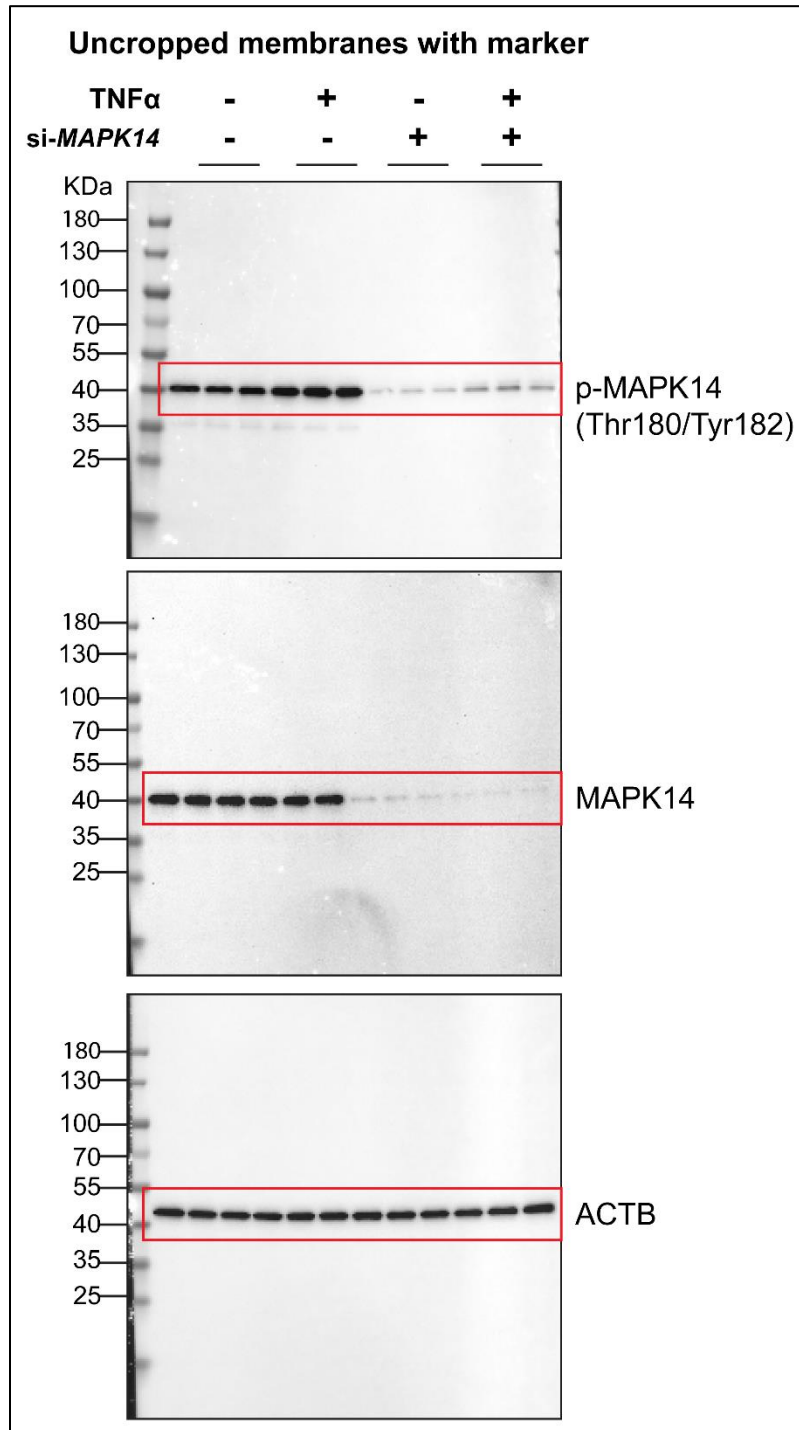

Fig. S5a

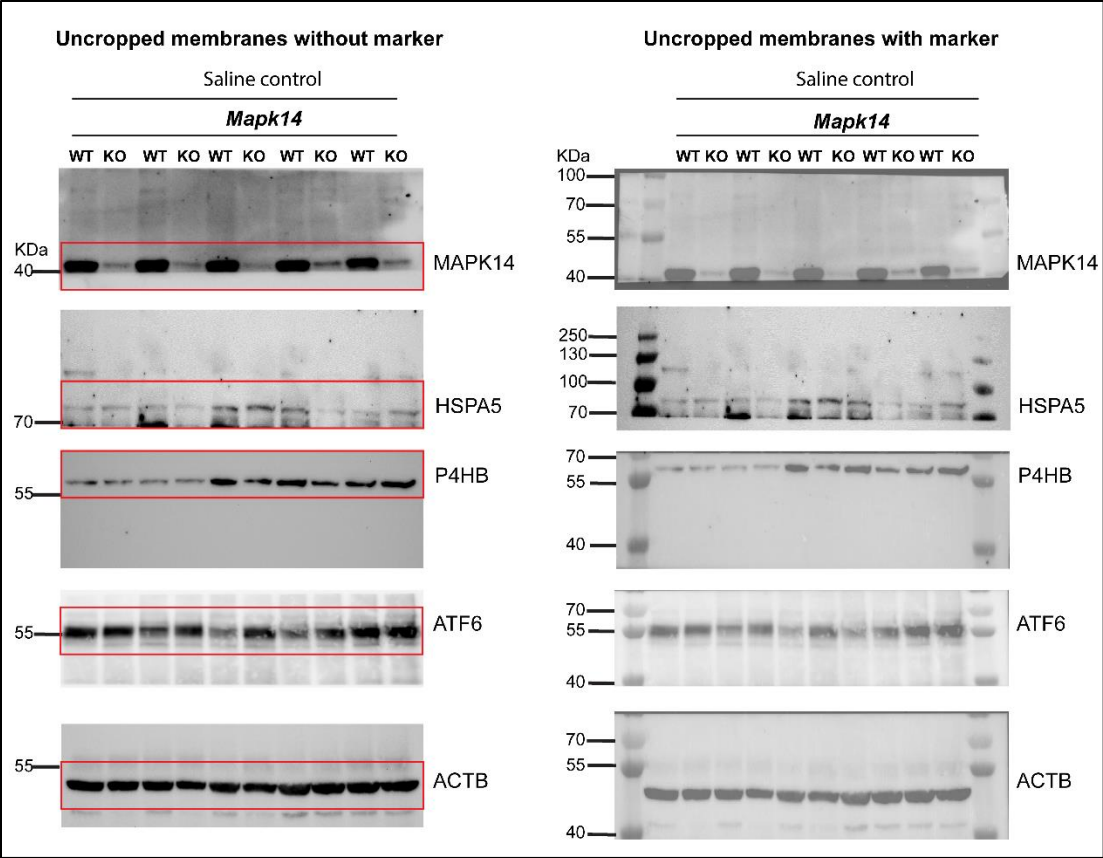

**Fig. S8a**

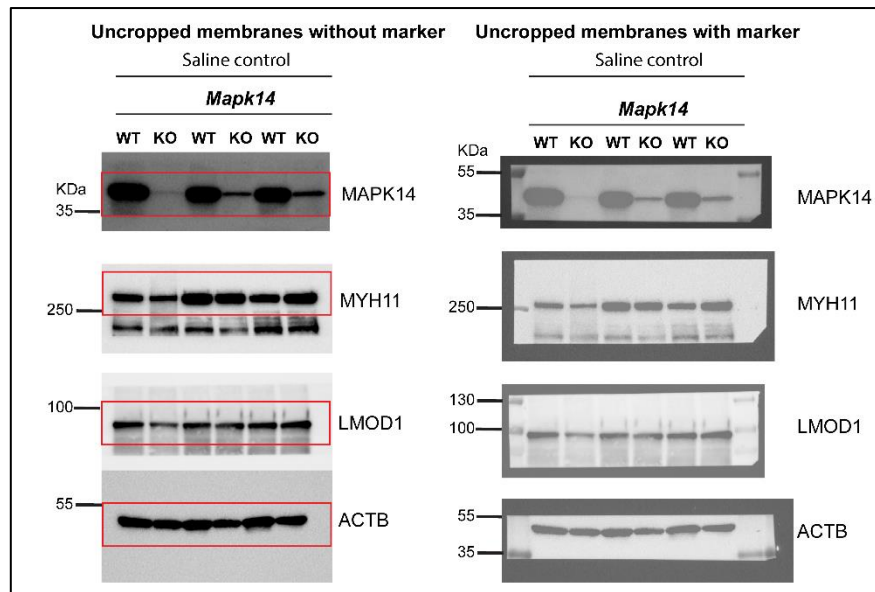

**Fig. S10a**

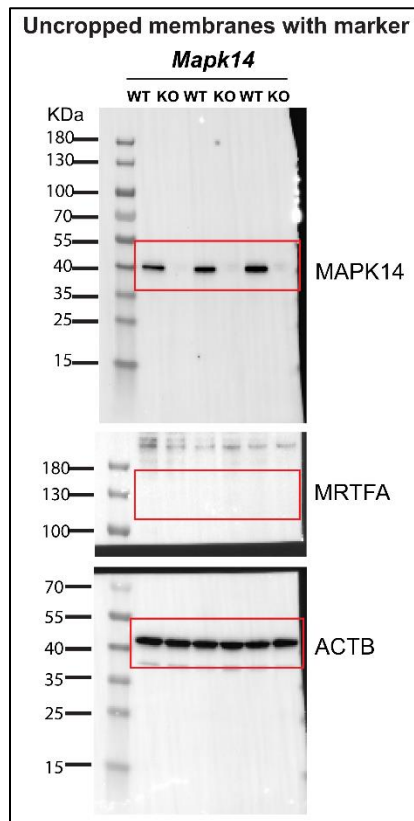

Supplement: Supplementary file 2 — Original western blots [file 41392_2025_2540_MOESM2_ESM.pdf]
